# Single‐Atom Ru Implanted on Co_3_O_4_ Nanosheets as Efficient Dual‐Catalyst for Li‐CO_2_ Batteries

**DOI:** 10.1002/advs.202102550

**Published:** 2021-10-20

**Authors:** Zheng Lian, Youcai Lu, Chunzhi Wang, Xiaodan Zhu, Shiyu Ma, Zhongjun Li, Qingchao Liu, Shuangquan Zang

**Affiliations:** ^1^ College of Chemistry Institute of Green Catalysis Zhengzhou University Zhengzhou 450001 P. R. China

**Keywords:** Co_3_O_4_ nanosheets arrays, discharge products, growth pathway, Li‐CO_2_ batteries, single‐atom catalysts

## Abstract

Li‐CO_2_ battery has attracted extensive attention and research due to its super high theoretical energy density and its ability to fix greenhouse gas CO_2_. However, the slow reaction kinetics during discharge/charge seriously limits its development. Hence, a simple cation exchange strategy is developed to introduce Ru atoms onto a Co_3_O_4_ nanosheet array grown on carbon cloth (SA Ru‐Co_3_O_4_/CC) to prepare a single atom site catalyst (SASC) and successfully used in Li‐CO_2_ battery. Li‐CO_2_ batteries based on SA Ru‐Co_3_O_4_/CC cathode exhibit enhanced electrochemical performances including low overpotential, ultra high capacity, and long cycle life. Density functional theory calculations reveal that single atom Ru as the driving force center can significantly enhance the intrinsic affinity for key intermediates, thus enhancing the reaction kinetics of CO_2_ reduction reaction in Li‐CO_2_ batteries, and ultimately optimizing the growth pathway of discharge products. In addition, the Bader charge analysis indicates that Ru atoms as electron‐deficient centers can enhance the catalytic activity of SA Ru‐Co_3_O_4_/CC cathode for the CO_2_ evolution reaction. It is believed that this work has important implications for the development of new SASCs and the design of efficient catalyst for Li‐CO_2_ batteries.

## Graphical abstract

A facile and eco‐friendly method to prepare single atom site catalysts is proposed, and the derived SA Ru‐Co_3_O_4_/CC is successfully applied to Li‐CO_2_ batteries. The doped single atom Ru sites have very high catalytic activity, which can not only improve the reaction kinetics but also optimize the growth pathway of the discharge products, thus comprehensively improving the electrochemical performance of the Li‐CO_2_ battery.

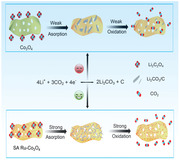

## 1 Introduction

Excessive use of fossil fuels has led to a rapid increase in CO_2_ emissions, leading to a series of serious environmental problems, of which global warming is the most urgent.^[^ ^1^, ^2^, ^3^ ^]^ In this case, attention is turning to technologies that can capture and use CO_2_, including electrochemical reduction, photoelectric chemical reduction, catalytic hydrogenation, catalytic reforming, metal‐CO_2_ battery, etc.^[^ ^4^, ^5^, ^6^, ^7^, ^8^, ^9^ ^]^ Among them, Li‐CO_2_ battery is regarded as one of the main candidates for new energy storage and conversion system due to its high theoretical energy density (1876 Wh kg^−1^) and the ability to fix CO_2_.^[^ ^8^, ^9^ ^]^ In addition, Li‐CO_2_ battery is considered to be a more promising priority in some high‐CO_2_ environments, such as underwater operations and Mars exploration.^[^ ^10^ ^]^ Though Li‐CO_2_ battery has been extensively studied and developed since it was first proposed in 2013,^[^ ^11^ ^]^ there are still many problems restricting its development, among which the main challenge is the hysteretic kinetics of the CO_2_ reduction reaction/CO_2_ evolution reaction (CRR/CER) process, which leads to unsatisfactory electrochemical performances including high overpotential, low capacity, pitiful reversible, and cycle stability of Li‐CO_2_ battery. Specifically, during the CRR process, the adsorption and activation of CO_2_ is difficult due to its thermodynamic stability.^[^ ^10^, ^12^ ^]^ Moreover, the solid products (Li_2_CO_3_ and C) will occupy a lot of space and active sites of the cathode catalyst, which limits the CRR kinetics to a large extent.^[^ ^13^ ^]^ While during the CER process, Li_2_CO_3_ is a thermodynamically stable wide‐bandgap insulator, which requires a higher charging potential (>4.3 V vs Li^+^/Li) to decompose. At such a high potential, the electrolyte and commonly used carbon cathode are also prone to decomposition.^[^ ^14^ ^]^ To make matters worse, due to adverse contact between discharge products and the active site, Li_2_CO_3_ is difficult to be completely decomposed during charging.^[^ ^15^ ^]^ This results in the accumulation of discharge products on the surface of the cathode, which prevents the mass transfer process and passivates the catalyst, further leading to problems such as high overpotential, sudden failure, and poor cycle stability. Therefore, the development of cathode catalysts with suitable pore structure and high catalytic activity is still a pressing matter in the development of Li‐CO_2_ batteries. To this end, various materials including carbon based materials,^[^ ^16^, ^17^ ^]^ transition‐metal oxides,^[^ ^18^, ^19^ ^]^ noble metals,^[^ ^20^, ^21^ ^]^ MOFs/COFs and their derivatives with rationally constructing hierarchical porous architectures,^[^ ^22^, ^23^, ^24^ ^]^ have been sprung up as efficient cathode catalysts to improve the performance of Li‐CO_2_ batteries. Actually, the implementation of these strategies can improve the performance of the battery to a certain extent. However, some unavoidable disadvantages, such as high cost, poor durability, relatively low capacity, poor cycle stability, and especially low utilization rate of active sites still exist. Hence, the development of cathode catalysts with high activity and durability under the premise of cost control is of great significance for Li‐CO_2_ batteries.

Recently reported single‐atom site catalysts (SASCs) as a hot research direction in the catalytic field has aroused wide attention and is even regarded as the “holy grail” of catalysts.^[^ ^25^, ^26^ ^]^ Compared with traditional nanoparticle catalyst, SASCs catalyst has many unique characteristics,^[^ ^27^ ^]^ such as high atomic utilization (AUE) (close to 100%) and maximizing accessible active sites, which can decrease the use of the metal and reduce the cost of the cathode. In addition, SASCs also have unique coordination and electron effects due to the interaction between the metal active sites and the supporter, which make SASCs performed extremely excellent in oxygen reduction reaction (ORR),^[^ ^28^ ^]^ oxygen evolution reaction (OER),^[^ ^29^ ^]^ CO_2_RR,^[^ ^30^ ^]^ hydrogen reduction reaction (HER),^[^ ^31^ ^]^ and other fields,^[^ ^25^, ^26^ ^]^ and some progress has also been made in the application of SASCs in Li‐CO_2_ batteries.^[^ ^32^, ^33^ ^]^ However, the reported SASCs supporters for Li‐CO_2_ batteries are carbon which is not stable in the battery system and the performance of battery needs to be further improved. In addition, understanding the deposition/decomposition process of the discharge products optimized by SASCs is an important role in determining the kinetics of the CRR and CER process; however, there are few studies in this aspect, especially in CER process. Most importantly, majority of reported SASCs synthesis processes tend to require harsh conditions and tedious process, which not only increase the preparation cost of SASCs but also bring great difficulties to their large‐scale synthesis and application.^[^ ^28^ ^]^ Therefore, it is of great significance to develop a simple and mild method to prepare SASCs for improving the electrochemical performance of Li‐CO_2_ batteries.

In this study, we propose a facile and eco‐friendly method to load Ru atoms onto Co_3_O_4_ nanosheets surface supported on carbon cloth (marked as SA Ru‐Co_3_O_4_/CC) by combining template replication and cation exchange strategy, which is successfully used as a dual‐catalyst for Li‐CO_2_ batteries. It is worth mentioning that the synthesis method only uses water as the solvent, and calcination conditions are easy to reach (350 °C and air atmosphere), which greatly reduces the cost and difficulty of synthesis of SASCs. Experimental results show that Co_3_O_4_ nanosheets array with 3D architecture possess large specific surface area and suitable pores to facilitate the mass transfer of electrolyte and reactants, and provide enough space to store the discharge products (Li_2_CO_3_ and C). While atomically dispersed Ru species as active sites tailored the nucleation behavior of discharge products, thus optimizing their final distribution, which is favor of subsequent decomposition during charging process. Additionally, density functional theory (DFT) simulation demonstrated that the introduced Ru atoms as the driving force center can significantly enhance the intrinsic affinity of the key intermediates (Li_2_C_2_O_4_), which is beneficial to CRR process. Meanwhile, the calculation of Co/Ru electron charge shows that Ru atoms also act as an electron‐deficient center, which is beneficial to the CER process of the battery. Benefiting from these merits, Li‐CO_2_ batteries based on SA Ru‐Co_3_O_4_/CC cathode exhibit enhanced electrochemical performances, including low overpotential, high specific capacity, and long cycle stability. We believe that this work can not only guide the design of efficient Li‐CO_2_ batteries but also have significant implications for the design and synthesis of SASCs.

## 2 Results and Discussion

**Figure 1:**
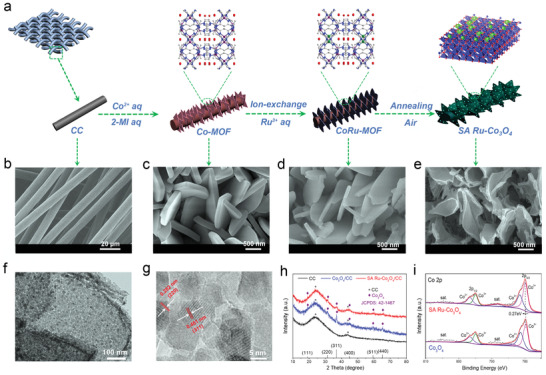
Structure characterization of SA Ru‐Co_3_O_4_/CC. a) Illustration of the synthesis process of SA Ru‐Co_3_O_4_/CC. b–e) SEM images of CC, Co‐MOF/CC, CoRu‐MOF/CC, and SA Ru‐Co_3_O_4_/CC. f) TEM image of SA Ru‐Co_3_O_4_. g) HRTEM image of SA Ru‐Co_3_O_4_. h) XRD patterns of CC, Co_3_O_4_/CC, and SA Ru‐Co_3_O_4_/CC. i) XPS spectra of Co 2p.

**Figure** **1**a depicts the synthesis strategy for the SA Ru‐Co_3_O_4_/CC cathode catalyst. Firstly, to grow Co‐MOF homogenously, the substrate of carbon cloth (CC) was treated by Ar plasma, and after an ingenious subsequent ion exchange and template replication process, the aimed cathode catalyst was obtained. Observing the whole synthesis process, all the solvents (only water) used are eco‐friendly and the preparation process is mild and facile (calcination temperature is 350 °C, air atmosphere), and hence this method has the potential of large‐scale synthesis and application.^[^ ^34^, ^35^ ^]^ The scanning electron microscope (SEM) images show the smooth carbon fiber with diameter of about 10 µm were woven with each other (Figure 1b), and then Co‐MOF is vertically and homogenously grown onto the skeleton of the CC (Co‐MOF/CC) without the help of any binder (Figure 1c and Figure S1a, Supporting Information). The SEM image of Co‐MOF/CC shows that the Co‐MOF presents triangular morphology, with a side length of ≈1.5 µm and a thickness of ≈180 nm (Figure 1c). As the similar coordination mode of Ru^3+^ and Co^2+^ for 2‐methylimidazole, the cation exchange process driven by the concentration is very easy. So it is easy to understand that Ru^3+^ in aqueous solution partially replaces Co metal nodes of Co‐MOF to generate CoRu‐MOF. X‐ray diffraction (XRD) spectra show that CoRu‐MOF and Co‐MOF have the same peak position, indicating that Ru^3+^ replaces Co^2+^, rather than forming new MOF or Ru ions adsorbed on the surface of Co‐MOF, as shown in Figure S2 (Supporting Information). In addition, the morphology of CoRu‐MOF is basically inherited from Co‐MOF, with only slight deformation and wrinkle appear on the surface (Figure 1d). Based on previous studies,^[^ ^36^ ^]^ it is supposed that this is due to the etching of MOF by H^+^ derived from the partial hydrolysis of Ru^3+^. To prove this, dilute hydrochloric acid was used to etch Co‐MOF/CC and a similar morphology to that of CoRu‐MOF was also found as shown in Figure S1b (Supporting Information). Finally, after annealing in the air, the ligand (2‐methylimidazole) in the CoRu‐MOF was removed and Co metal is oxidized into Co_3_O_4_, while the Ru species in CoRu‐MOF coordinates with O atoms in Co_3_O_4_, thus being anchored on the surface of Co_3_O_4_ nanosheet. The obtained SA Ru‐Co_3_O_4_ presented a slightly curled elliptical morphology (Figure 1e), while the Co_3_O_4_ almost inherits the morphology of its precursor as shown in Figure S3 (Supporting Information). The synthesized SA Ru‐Co_3_O_4_/CC with 3D architecture acting as cathode is favorable for gas and electrolyte transport.^[^ ^12^, ^18^ ^]^ In addition, the considerable surface area could provide enough space for the storage of discharge products (Figure S4, Supporting Information). It is worth noting here that the surface area and pore volume/size of SA Ru‐Co_3_O_4_ and Co_3_O_4_ are almost identical, indicating that the difference in their catalytic properties is derived from their essential catalysis.

Transmission electron microscope (TEM) images confirmed that the SA Ru‐Co_3_O_4_ nanosheets and Co_3_O_4_ nanosheets were composed by many nanoparticles with sizes ranging from 10 to 20 nm, as shown in Figure 1f and Figure S5 (Supporting Information). In addition, the images of the selected area electron diffraction (SAED) analysis of SA Ru‐Co_3_O_4_ and Co_3_O_4_ nanosheets show diffraction rings associated only with Co_3_O_4_ (Figure S6, Supporting Information), demonstrating that the introduction of Ru atoms hardly changes the crystal structure of the supporter. High‐resolution TEM (HRTEM) image (Figure 1g and Figure S7, Supporting Information) shows the lattice fringes of the SA Ru‐Co_3_O_4_ and Co_3_O_4_ nanosheets, which can be ascribed to the (311) and (220) planes of cubic spinel Co_3_O_4_ (JCPDS card No. 42‐1467). To identify the distribution of Ru species on SA Ru‐Co_3_O_4_, elemental mapping was performed with energy‐dispersive X‐ray spectrometry (EDS) as shown in Figures S8 and S9 (Supporting Information), it could be found that Ru elements were uniformly distributed on the SA Ru‐Co_3_O_4_. Figure 1h shows the XRD pattern of CC, Co_3_O_4_/CC, and SA Ru‐Co_3_O_4_/CC, only the peaks of CC and Co_3_O_4_ were found on the XRD curves of SA Ru‐Co_3_O_4_/CC, without any peaks related to Ru species,^[^ ^37^ ^]^ and this result was confirmed by Raman spectra (Figure S10, Supporting Information). Notably, with the introduction of Ru atoms, the peaks intensity of both XRD and Raman were weakened, which may be derived from the two aspects: The incorporation of Ru atoms partially destroy the order of Co_3_O_4_ crystal structure, making it less crystallinity; and the vibration intensity of the original Co_3_O_4_ crystal is reduced with the introduction of Ru heteroatom.^[^ ^38^, ^39^ ^]^

X‐ray photoelectron spectroscopy (XPS) analysis is used to preliminarily study the surface chemical status of SA Ru‐Co_3_O_4_/CC samples. The peak‐fitting analysis of Co 2p spectrum shows that there are two chemical states (Figure 1i), corresponding to Co^2+^ at 781.6 and 797.2 eV and Co^3+^ at 780.1 and 795.7 eV.^[^ ^37^ ^]^ The high‐resolution O 1s spectrum can be deconvoluted into four peaks (Figure S11, Supporting Information), namely, the metal‐O peak at 529.7 eV, oxygen vacancy peak at 531.1 eV, metal‐O‐H peak at 532.4 eV, and the adsorbed oxygen species peak at 534.0 eV.^[^ ^36^ ^]^ Because the XPS peak positions of Ru 3d and C 1s are basically overlapping, and SA Ru‐Co_3_O_4_ nanosheets grow on CC, it is relatively difficult to analyze Ru element. Even so, a relatively weak peak at 281.8 eV was isolated from the Ru 3d and C 1s spectra, which can be attributed to the Ru species in the oxidized state,^[^ ^35^ ^]^ as shown in Figure S12 (Supporting Information). This also confirms the successful incorporation of Ru species.

**Figure 2:**
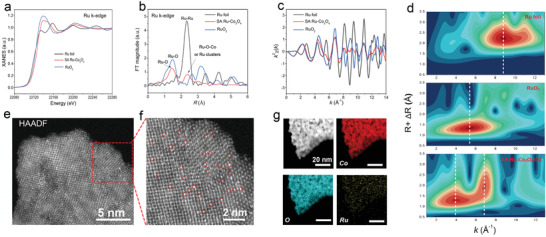
Structure characterization of Ru single atom in SA Ru‐Co_3_O_4_/CC. a,b) Ru K‐edge XANES and EXAFS for SA Ru‐Co_3_O_4_, RuO_2_, and Ru foil. c) Fourier‐transformed magnitudes of Ru K‐edge EXAFS spectra in K space for SA Ru‐Co_3_O_4_. d) WT for the k^2^‐weighted EXAFS signal. e,f) HAADF‐STEM images of SA Ru‐Co_3_O_4_. g) HAADF‐STEM image and related elemental mapping images of SA Ru‐Co_3_O_4_.

To further investigate the electron state and local structure of SA Ru‐Co_3_O_4_, X‐ray absorption fine structure (XAFS) spectroscopies are conducted to characterize the electron state and coordination configurations of Ru in SA Ru‐Co_3_O_4_. Firstly, for identifying the 3D arrangement of Ru atoms at higher sensitivity, X‐ray absorption near‐edge structure (XANES) analysis is applied (**Figure** **2**a). The notable difference of XANES profiles between Ru foil and SA Ru‐Co_3_O_4_ clearly excludes the Ru‐Ru interaction in the as‐prepared SA Ru‐Co_3_O_4_. Due to the absorption edge energy is proportional to the oxidation states, highly valent cations show stronger binding energy.^[^ ^27^ ^]^ Based on the line position (around 22 120 eV) of SA Ru‐Co_3_O_4_/CC in Figure 2a, the valence state of Ru in SA Ru‐Co_3_O_4_/CC is even higher than that in RuO_2_ (+4). It should be taken into account that Ru atoms are mainly located on the surface of the precursor Co‐MOF nanosheets rather than inside, which means that Ru atoms may be fully oxidized to a higher valence state during the calcination process. And it is worth mentioning that a similar situation has appeared in some similar reports.^[^ ^34^, ^35^ ^]^ Then, to verify the local environment of Ru species, the extended XAFS (EXAFS) spectra of Ru K‐edge of SA Ru‐Co_3_O_4_ were analyzed. The Fourier‐transformed EXAFS (FT‐EXAFS) curve of the Ru k‐edge of SA Ru‐Co_3_O_4_ shows a distinct peak at 1.45 Å (Figure 2b), located close to the Ru—O bond in commercial RuO_2_ (1.48 Å), which should be assigned to the Ru—O bond in SA Ru‐Co_3_O_4_.^[^ ^40^ ^]^ Note that, there is a small peak at around 2.3 Å, which is slightly bigger than the peak of the Ru—Ru bond in the Ru foil. According to previous reports, this may be due to the Ru—Ru bond in the small Ru cluster, or the bond between Co and Ru (representing Ru‐O‐Co in the crystal structure),^[^ ^34^ ^]^ we will further explore the attribution of this peak through subsequent characterization methods. The coordination sphere of the atomically dispersed centers is further quantified by least‐squares EXAFS curvefitting analyses (Figure 2c and Figure S13, Supporting Information). The results confirmed that the Ru bonding coordination number in the first coordination spherical shell were 5.0 ± 0.3, similar to the Co atoms at octahedral points in Co_3_O_4_, which further proved that Ru atoms generated the CoRu‐MOF precursor by replacing the Co nodes in the Co‐MOF. The above results suggest that the decorated Ru atoms keep the nearly identical configuration to that of Co atoms in Co_3_O_4_. In the end, wavelet transform (WT) simulations have also been conducted for interpreting a radial distance resolution in the K space, as shown in Figure 2d. By comparing with Ru foil and commercial RuO_2_, it is found that there are two obvious WT signals in Ru‐Co_3_O_4_, their positions are, respectively, around 3.9 and 6.8 Å^−1^. Among them, the WT intensity maximum near 3.9 Å^−1^ arising from the Ru—O coordination is well resolved at 1.0–2.0 Å for SA Ru‐Co_3_O_4_. While the signal near 6.8 Å^−1^ is different from the signal value of the maximum intensity of Ru foil (≈8.8 Å^−1^), then the WT signal should be assigned to the Ru—Co bond (representing Ru‐O‐Co in the crystal structure) or small Ru clusters.^[^ ^34^ ^]^ In addition, the detailed parameters of SA Ru‐Co_3_O_4_ measured by XAFS are shown in Table S1 (Supporting Information).

To further verify the existence of atomically dispersed Ru atoms on SA Ru‐Co_3_O_4_, SA Ru‐Co_3_O_4_ samples were analyzed on the atomic scale by using atomic resolution aberration‐corrected high‐angle annular dark‐field scanning TEM (HAADF‐STEM). The results demonstrated that numerous bright spots (Ru atoms) are evenly distributed on the substrate; and some bright spots are close to each other, which means that small Ru clusters may also exist on SA Ru‐Co_3_O_4_ nanosheets, as shown in Figure 2e,f. In addition, energy‐dispersive X‐ray spectroscopy (EDXS) images indicate that the Ru atoms are distributed uniformly over the entire SA Ru‐Co_3_O_4_ nanosheets (Figure 2g), and the weight percentage of Ru element on SA Ru‐Co_3_O_4_ nanosheets is 3.05 wt%, which is close to the inductively coupled plasma‐optical emission spectrometer (ICP‐OES) test results (3.16 wt%).

**Figure 3:**
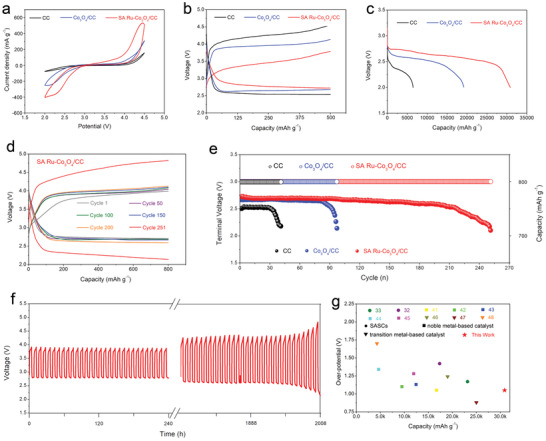
Electrocatalytic performance evaluation of the various cathodes in Li‐CO_2_ batteries. a) Cyclic voltammetry (CV) curves of CC, Co_3_O_4_/CC, and SA Ru‐Co_3_O_4_/CC based batteries, the scan rate is 0. 1 mV s^−1^. b) Initial discharge/charge profiles of CC, Co_3_O_4_/CC, and SA Ru‐Co_3_O_4_/CC at 100 mA g^−1^ under the limited discharge/charge capacities of 500 mAh g^−1^. c) Full discharge profiles of CC, Co_3_O_4_/CC, and SA Ru‐Co_3_O_4_/CC tested at 100 mA g^−1^. d) Discharge/charge profiles of SA Ru‐Co_3_O_4_/CC cathode at 200 mA g^−1^. e) Cycling performance comparison of CC, Co_3_O_4_/CC, and SA Ru‐Co_3_O_4_/CC, at 200 mA g^−1^. f) Cycling performance of SA Ru‐Co_3_O_4_/CC cathode at 200 mA g^−1^. g) Comparison of overpotential and full discharge capacity between SA Ru‐Co_3_O_4_/CC cathode based battery and some previously reported Li‐CO_2_ batteries (the numbers represent the serial numbers of references).

Li‐CO_2_ batteries based on CC, Co_3_O_4_/CC, and SA Ru‐Co_3_O_4_/CC cathodes were assembled and their various electrochemical performances were tested. **Figure** **3**a presents the cyclic voltammetry (CV) response of batteries at a constant scan rate of 0.1 mV s^−1^ within 2.0–4.5 V. It is noted that battery with SA Ru‐Co_3_O_4_/CC cathode exhibits highest reduction onset potential and lowest oxidation onset potential, suggesting highest catalytic activity of SA Ru‐Co_3_O_4_/CC cathode compared to those of CC and Co_3_O_4_/CC cathodes. Figure 3b displays the first discharge–charge profiles of Li‐CO_2_ batteries with the different cathodes at a cut‐off capacity of 500 mAh g^−1^ with a constant current density of 100 mA g^−1^. Obviously, the battery with SA Ru‐Co_3_O_4_/CC cathode shows the much decreased overpotential (1.05 V) compared to batteries with CC (1.45 V) and Co_3_O_4_/CC (1.98 V) cathodes. And Li‐CO_2_ batteries with SA Ru‐Co_3_O_4_/CC cathode still maintain lowest overpotential even at high current density and large restrict capacity (Figures S14 and S15, Supporting Information). In addition, the rate performance of Li‐CO_2_ batteries was evaluated in the current density range of 50–500 mA g^−1^ (Figure S16, Supporting Information), and it was found that Li‐CO_2_ battery based on SA Ru‐Co_3_O_4_/CC cathode exhibited the highest discharge platform at various current densities. The improved reduction/oxidation kinetics of SA Ru‐Co_3_O_4_/CC cathode may be derived from several synergies: the introduced atomically dispersed Ru atoms facilitate the adsorption of intermediates and thus promoting reduction (discharge) kinetics; atomically dispersed Ru atoms as electron‐deficient centers promote the transfer of electrons in the oxidation (charge) process; and the deposition behavior and morphology of the discharge products were well tailored (vide infra), which is beneficial to the subsequent oxidation charging process. The tailored discharge products encouraged us to investigate the discharge capacity of batteries. Figure 3c displays the discharge curves of batteries with different cathodes at the current density of 100 mA g^−1^ and cut‐off voltage of 2.0 V. Unexpectedly, battery with SA Ru‐Co_3_O_4_/CC cathode exhibits a higher discharge capacity of 30 915 mAh g^−1^ than those with the CC (6468 mAh g^−1^), Co_3_O_4_/CC (19 228 mAh g^−1^) cathodes, even at a higher current density of 300 mA g^−1^, the discharge capacity of the SA Ru‐Co_3_O_4_/CC cathode can still reach 16 510 mAh g^−1^ (Figure S17a, Supporting Information), which is among one of the largest discharge capacity of Li‐CO_2_ battery reported so far. To rule out that the discharge/charge capacity of Li‐CO_2_ batteries is due to the parasitic reaction caused by electrolyte decomposition, a deep discharge/charge test on batteries based on different cathodes in Ar atmosphere was conducted. The results show that Li‐CO_2_ batteries based on three different cathodes all exhibit negligible discharge/charge capacity in Ar atmosphere, which indicates that the discharge capacity of our Li‐CO_2_ batteries is mainly from the electrochemical reaction of reaction gas CO_2_ and Li^+^, and the charge capacity is mainly from the decomposition of discharge products (Figure S17b, Supporting Information). To highlight the catalytic activity of Ru single atom, Co_3_O_4_/CC cathode modified by RuO_2_ nanoparticles (marked as RuO_2_‐Co_3_O_4_/CC) was also prepared as shown in Figure S18 (Supporting Information). The electrochemical performances of the synthesized RuO_2_‐Co_3_O_4_/CC cathode were tested as shown in Figure S19 (Supporting Information). It can be seen that although the electrochemical performances of RuO_2_‐Co_3_O_4_/CC are improved compared with that of pristine Co_3_O_4_, the catalytic activity of RuO_2_‐Co_3_O_4_/CC is still lower than that of SA Ru‐Co_3_O_4_/CC, further indicating the advantage of single atom catalyst in the field of Li‐CO_2_ batteries.

Cyclic stability is an important index to evaluate Li‐CO_2_ batteries performance. The discharge/charge profiles of batteries with different cathodes are shown in Figure 3d and Figure S20 (Supporting Information), and their corresponding discharge terminal voltages were given in Figure 3e. The cycling were tested by controlling the discharge/charge depth of 800 mA h g^−1^ with a current density of 200 mA g^−1^. Obviously, compared with CC (41 cycles) and Co_3_O_4_/CC (97 cycles) cathodes, the cycling stability of SA Ru‐Co_3_O_4_/CC cathode (251 cycles) was significantly improved, which might be ascribed to the increased catalytic activity and optimized discharge products growth pathway caused by atomically dispersed Ru atoms (vide infra). Their corresponding time–voltage profiles are illustrated in Figure 3f, it could be found that battery with SA Ru‐Co_3_O_4_/CC cathode could sustain a stable discharge and charge potentials and operate more than 2000 h, and this is superior to that of battery with CC (328 h) and Co_3_O_4_/CC (776 h) cathodes, respectively. Li‐CO_2_ battery with SA Ru‐Co_3_O_4_/CC cathode can maintain comparable cycle stability even under deep charge/discharge cycles as shown in Figure S21 (Supporting Information). To further highlight the advantages of SA Ru‐Co_3_O_4_/CC cathode catalyst, the performances of SA Ru‐Co_3_O_4_/CC cathode catalyst were compared with some previously reported Li‐CO_2_ battery cathode catalysts,^[^ ^32^, ^33^, ^41^, ^42^, ^43^, ^44^, ^45^, ^46^, ^47^, ^48^ ^]^ as shown in Figure 3g and Table S2 (Supporting Information). Although the overpotential of Li‐CO_2_ batteries based on SA Ru‐Co_3_O_4_/CC cathodes is not shocking enough, but its properties are still better than that of most reported Ru‐based catalysts and SASCs in Li‐CO_2_ batteries system. Furthermore, considering that the load of precious metal Ru in SA Ru‐Co_3_O_4_/CC is much lower than other reported Ru‐based nanoparticles, such an overpotential is acceptable and competitive. In general, the electrochemical performances of Li‐CO_2_ battery with SA Ru‐Co_3_O_4_/CC cathode are superior to report in most literatures.

**Figure 4:**
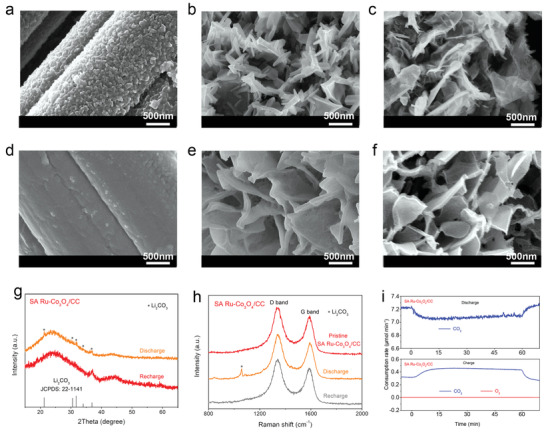
Reversibility of the various cathodes in Li‐CO_2_ batteries. a–c) SEM images of CC, Co_3_O_4_/CC, and SA Ru‐Co_3_O_4_/CC cathodes after discharged, current density: 200 mA g^−1^, cut‐off capacity: 800 mAh g^−1^. d–f) SEM images of CC, Co_3_O_4_/CC, and SA Ru‐Co_3_O_4_/CC cathodes after recharged. g,h) XRD patterns and Raman spectra of Li‐CO_2_ battery with a SA Ru‐Co_3_O_4_/CC cathode under the discharged and recharged states, respectively. i) DEMS test of Li‐CO_2_ battery with a SA Ru‐Co_3_O_4_/CC cathode during discharging and charging, current density: 200 µA, cut‐off capacity: 200 µAh.

To further reveal the mechanism associated with atomically dispersed Ru atoms in Li‐CO_2_ battery, the evolution of the morphology and composition of the discharged and recharged cathodes was investigated. Under the current density of 200 mA g^−1^ and the cut‐off capacity of 800 mAh g^−1^, SEM technique was used to observe the pristine, discharged, and charged CC, Co_3_O_4_/CC and SA Ru‐Co_3_O_4_/CC cathodes as shown in **Figure** **4**a–f and Figure S22 (Supporting Information). It can be observed that the discharge products on CC, Co_3_O_4_/CC, and SA Ru‐Co_3_O_4_/CC cathodes present three different morphologies, which are granular, massive shape, and thin flaky shape, respectively, as shown in Figure 4a–c. According to previous reports, this may be caused by different growth pathway of the discharge products on different cathodes, while the growth pathway and morphology of the discharge products have a great impact on the performance of the Li‐CO_2_ batteries.^[^ ^10^, ^41^ ^]^ This phenomenon will be discussed in detail in the following sections by conjunction with the results of DFT calculations. After charging, there are still a small amount of small granular discharge products remaining on the surface of the CC cathode, while the discharge products on the Co_3_O_4_/CC and SA Ru‐Co_3_O_4_/CC cathodes are both decomposed (Figure 4d–f). To investigate the rechargeability of three different cathodes, after running for five cycles, the cathodes were extracted and found that there was a large amount of undecomposed discharge products accumulate on the CC cathode and a small amount of residual discharge products remained on product on the Co_3_O_4_/CC cathode (Figures S23 and S24, Supporting Information), while the SA Ru‐Co_3_O_4_/CC cathode electrode still maintained its pristine morphology (Figure S25a, Supporting Information). Even after 30 cycles, there is only a small amount of residual discharge products on the surface of SA Ru‐Co_3_O_4_/CC cathode (Figure S25b, Supporting Information), demonstrating that the SA Ru‐Co_3_O_4_/CC cathode has excellent rechargeability durability. And HAADF‐STEM image also showed that the Ru atoms on the SA Ru‐Co_3_O_4_ nanosheets were still atomically dispersed after 5 and 30 cycles (Figure S25c,d, Supporting Information). In addition, after running 5 and 30 cycles, the XRD and Raman peaks of the SA Ru‐Co_3_O_4_/CC cathode barely changed, which indirectly proved the stability of the single atom (Figure S26, Supporting Information). Corresponding XRD and Raman spectra also confirmed the formation and decomposition of Li_2_CO_3_ in the discharge products (Figure 4g,h; Figures S27 and S28, Supporting Information). In addition, Raman spectra analysis showed that the *I*_D_/*I*_G_ ratio of the discharged cathodes was significantly higher than that of the pristine and the recharged cathodes, indicating that amorphous carbon is generated in discharge process and decomposed in subsequent charge process.

To further investigate the operating mechanism of Li‐CO_2_ batteries based on different cathodes, in situ differential electrochemical mass spectrometry (DEMS) was used to monitor the consumption and production of CO_2_ under a current of 0.2 mA with a cut‐off capacity of 0.2 mA h, as shown in Figure 4i and Figure S29 (Supporting Information). According to previous reports, the general accepted discharge and charge reactions are as follows:

(1) $$4Li^{+}+3CO_{2}+4e^{-}⇌2Li_{2}CO_{3}+C$$

(2) $$2Li_{2}CO_{3}+C⇌4Li^{+}+3CO_{2}+4e^{-}$$

According to above equation, the reversible Li‐CO_2_ battery is a four‐electron transfer process, and the theoretical molar ratio of electron transfer to CO_2_ generation/consumption (e^−^/CO_2_) is about 1.33. In addition to the above paths, some other possible charging reaction paths were also proposed in recently reported literatures, such as Li_2_CO_3_ self‐decomposition and O_2_^•−^ mediated Li_2_CO_3_ self‐decomposition according to Equations (3) and (4), and the corresponding theoretical e^−^/CO_2_ values are 1.5 and 2.0, respectively^[^ ^8^, ^10^ ^]^

(3) $$2Li_{2}CO_{3}\to 2CO_{2}+O_{2}+4Li^{+}+4e^{-}$$

(4) $$2Li_{2}CO_{3}\to 2CO_{2}+{O_{2}}^{•-}+4Li^{+}+3e^{-}$$

However, based on the results of DEMS, no O_2_ was detected as gas product during the charging process and the Eq (3) was was excluded. The experimental results show that the e^−^/CO_2_ ratio of Li‐CO_2_ battery based on SA Ru‐Co_3_O_4_/CC cathode is close to 1.33 during the discharge (1.37) and charge (1.39) process. Combined with the results of XRD pattern and Raman spectra, it is suggested that the discharge and charge reaction of Li‐CO_2_ battery based on SA Ru‐Co_3_O_4_/CC cathode may be in accordance with Equation (1).^[^ ^15^ ^]^ For Li‐CO_2_ batteries based on CC and Co_3_O_4_/CC cathode, the ratio of e^−^/CO_2_ during discharge is 1.47 and 1.56, respectively. However, during charging, the e^−^/CO_2_ ratio of Li‐CO_2_ batteries based on CC and Co_3_O_4_/CC cathode is 1.52 and 1.79, respectively, which is significantly different from the theoretical value (1.33). We supposed that this may be attributed to the following two reasons: During the charging process, CC and Co_3_O_4_/CC cathodes are difficult to promote the reaction between Li_2_CO_3_ and carbon species due to their insufficient catalytic activity, leading to a part of Li_2_CO_3_ is decomposed through O_2_^•−^ mediated self‐decomposition pathway; and the higher charging potential leads to the decomposition of the electrolyte and cathode, resulting in a series of unknown parasitic reactions. This also explains why Li‐CO_2_ battery based on SA Ru‐Co_3_O_4_/CC cathode shows better cycle stability.

**Figure 5:**
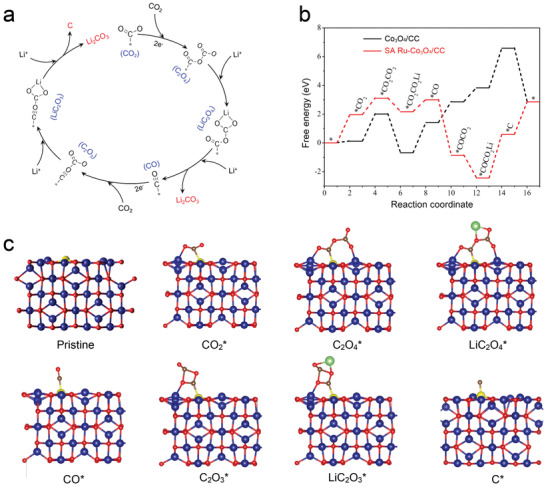
Study on CRR process mechanism of Li‐CO_2_ batteries. a) Simulation path and possible reaction intermediates of Li‐CO_2_ battery during discharge. b) Schematic Gibbs free energy diagrams of formation and decomposition of the discharge products during discharging. c) Simulation of reactants and reaction intermediates on the “SA Ru‐Co_3_O_4_” active sites during discharge.

To obtain a clear mechanistic understanding of the high performance for the SA Ru‐Co_3_O_4_/CC cathode toward CO_2_ reduction and evolution, DFT calculations were carried out to simulate the electronic structures of various active sites.^[^ ^32^, ^33^ ^]^ To illustrate more precisely the role of atomically dispersed Ru atoms in this system, here we simulated only a series of reactions in a battery using Co_3_O_4_/CC and SA Ru‐Co_3_O_4_/CC cathodes. According to above analysis combined with the previous reports,^[^ ^10^, ^33^ ^]^ the process shown in **Figure** **5**a was used as the reaction formula of the Li‐CO_2_ battery for simulations. Figure 5b shows the free energy diagram of discharge for Co_3_O_4_/CC and SA Ru‐Co_3_O_4_/CC cathodes. During the discharge process, their rate‐determining step is the formation of *COCO_2_Li intermediate (Figure 5b). According to the calculation, the theoretical discharge platforms of Co_3_O_4_/CC and SA Ru‐Co_3_O_4_/CC cathodes are 2.75 and 3.03 V, respectively. The side views of the configuration of different intermediates on the reactive sites of SA Ru‐Co_3_O_4_ and Co_3_O_4_ during the discharge process are shown in Figure 5c and Figure S30 (Supporting Information). Obviously, both the calculated and experimental results show that the discharge potential of Li‐CO_2_ battery based on SA Ru‐Co_3_O_4_/CC cathode is higher than that of with Co_3_O_4_/CC cathode.

**Figure 6:**
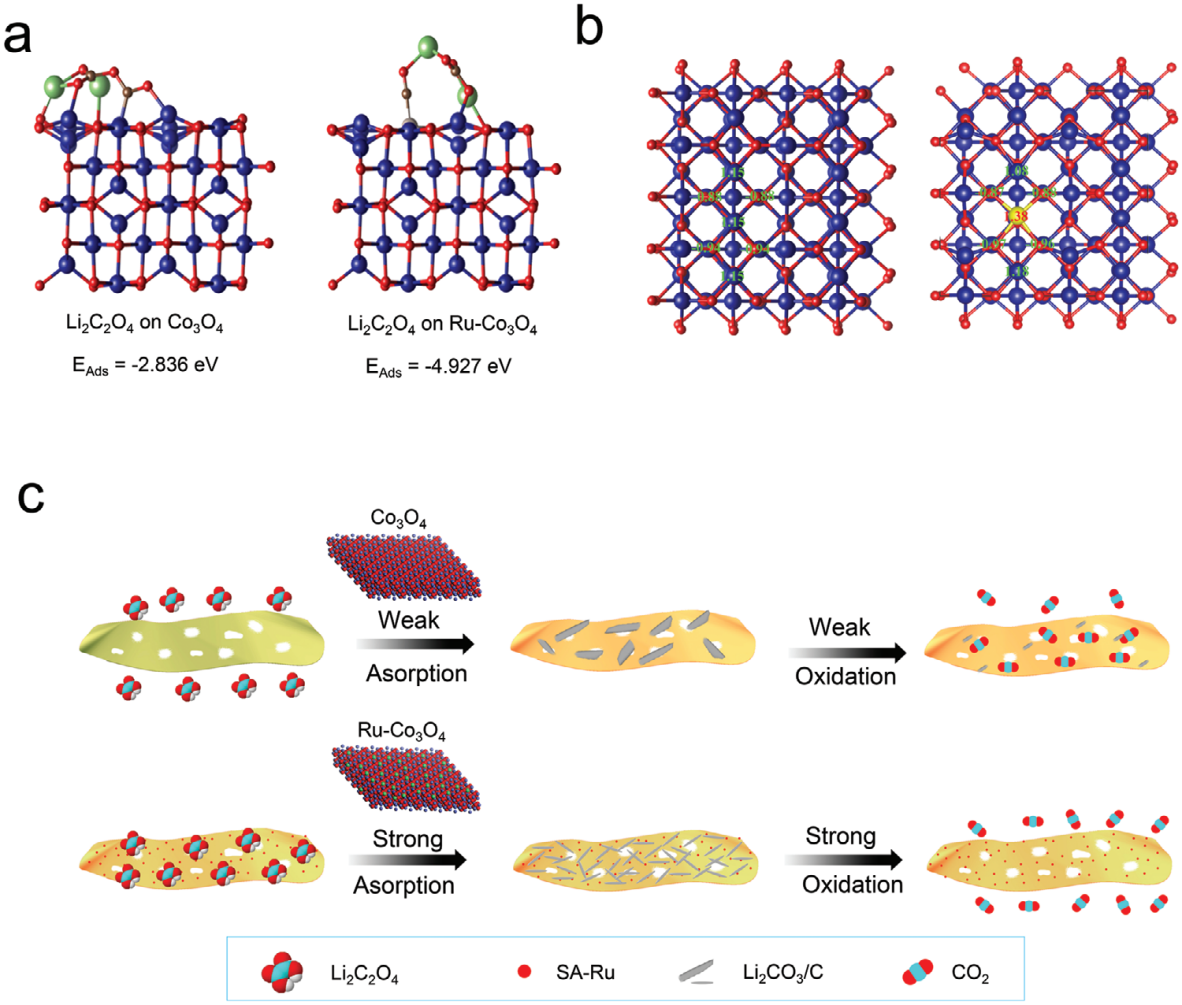
Study on the growth pathway of discharge product in Li‐CO_2_ battery. a) Adsorption energy of reaction intermediate Li_2_C_2_O_4_ on Co_3_O_4_ and SA Ru‐Co_3_O_4_ active sites. b) Bader charge distribution on Co_3_O_4_ and SA Ru‐Co_3_O_4_. c) Schematic illustrations of the working mechanism for the Co_3_O_4_/CC and SA Ru‐Co_3_O_4_/CC cathodes.

The morphology and distribution of discharge products depended on their growth pathway, which is important for the performance of the battery,^[^ ^18^, ^23^ ^]^ while there are few related reports in Li‐CO_2_ battery field. In the Li‐CO_2_ battery system, similar to Li‐O_2_ battery, there are two recognized growth pathways of discharge products: solvation‐mediated growth pathway and surface‐adsorption growth pathway.^[^ ^49^, ^50^, ^51^ ^]^ In our previous report, the different growth pathway of discharge products in Li‐CO_2_ batteries was initially explained by calculating the adsorption energy of different active sites for discharge product Li_2_CO_3_.^[^ ^41^ ^]^ Specifically, because the Li_2_CO_3_ has certain solubility, during its growth period, if the binding energy of Li_2_CO_3_ to the active sites is weaker; it will preferentially nucleate and grow in the electrolyte, and then accumulation on the cathode according to the solvation‐mediated growth pathway. On the contrary, when the binding energy of Li_2_CO_3_ to active sites is stronger, it is more inclined to form a layer of Li_2_CO_3_ on the cathode surface through the surface‐adsorption growth pathway. However, in combination with the above analysis and current literature reports, it is incomplete to use Li_2_CO_3_ as adsorbent to study the reaction pathway, since it is formed in two steps during the reaction process (Figure 5a). Some key reaction intermediates such as Li_2_C_2_O_4_ is more suitable as candidate and have been well accepted as intermediates in the Li‐CO_2_ batteries CRR process.^[^ ^8^, ^10^, ^22^ ^]^ It is supposed that the binding energy of the active site for the reaction intermediates may also affect the growth pathway of the discharge products. Hence, the adsorption energies of reactant CO_2_, intermediate Li_2_C_2_O_4_ and discharge product Li_2_CO_3_ at the active sites of Co_3_O_4_ and SA Ru‐Co_3_O_4_ were calculated, repectively, and the calculation results are shown in Table S3 (Supporting Information). According to the change of bond length and bond angle of CO_2_, compared with the active site SA Ru‐Co_3_O_4_, the active site Co_3_O_4_ is easier to adsorb and activate CO_2_ (Tables S3 and S4, Supporting Information). In addition, the adsorption energy of the active site Co_3_O_4_ and SA Ru‐Co_3_O_4_ for the discharge product Li_2_CO_3_ is almost the same (Figure S31, Supporting Information). In spite of this, the adsorption energy of the active site SA Ru‐Co_3_O_4_ for the key reaction intermediate Li_2_C_2_O_4_ was −4.927 eV, which is more negative than that of Co_3_O_4_ (−2.836 eV) as shown in **Figure** **6**a. It is believe that the SA Ru‐Co_3_O_4_/CC cathode can promote the surface‐mediated reaction path of discharge products by enhancing the binding energy to the key reaction intermediate Li_2_C_2_O_4_. In addition, as encouraged by plentiful active sites triggered by atomically dispersed Ru atoms, homogeneously distributed discharge products were formed on the SA Ru‐Co_3_O_4_/CC cathode, which further verifies the SEM results. The homogeneously distributed discharge products increase the contact area with the cathode, which is conducive to the transfer of electrons and Li^+^ in the charging process. As a benefit, the high charge overpotential and parasitic reaction caused by high voltage can be greatly reduced. In addition, the uniform distribution of discharge products can alleviate the cathode volume effect to a large extent, which is beneficial to improve its stability. As for Li‐CO_2_ battery based on Co_3_O_4_/CC cathode, the growth of discharge products tended to be solvation‐mediated growth pathway according to the calculation results of adsorption energy, which eventually leads to the generation of massive shape discharge products.

Generally, the adsorption/activation of reactants and intermediates as well as the regulation of the reaction path of the battery caused by catalyst can greatly improve the performances of Li‐CO_2_ batteries. However, the key factor affecting the performance of batteries, especially the round‐trip efficiency, lies more in their charging process, that is, the oxidation process of discharge products (CO_2_ evolution reaction). How does the catalyst essentially promote the oxidation process is the key to understanding the reaction mechanism of the battery and improving its performance. However, there has been little research in this area. It is well known that the doping of heterogeneous atoms will regulate the charge distribution on the surface of the supporter, and then change its catalytic properties. Inspired by this concept, The Bader charge of Co and Ru sites on the surface of Co_3_O_4_ and SA Ru‐Co_3_O_4_, respectively, was calculated as shown in Figure 6b. The results show that the Ru site on SA Ru‐Co_3_O_4_ has a more positive charge than that of octahedral Co site on Co_3_O_4_. According to previous reports, the doped metal atoms as electron‐deficient centers can improve the catalytic activity for the oxidation process,^[^ ^52^, ^53^, ^54^, ^55^, ^56^, ^57^ ^]^ which is beneficial to reduce the charging overpotential of the battery. In conclusion, heterogeneous Ru atoms as the active centers not only enhance the CRR process of the battery, optimize the morphology and growth pathway of the discharge products, but also play a positive role in the battery charging process. All these results indicate that the synthesized SA Ru‐Co_3_O_4_ is an efficient dual catalyst for Li‐CO_2_ batteries.

To show how different cathodes tailor the growth pathway of discharge products, a schematic diagram of the discharge process of Li‐CO_2_ batteries based on Co_3_O_4_/CC and SA Ru‐Co_3_O_4_/CC cathodes is shown in Figure 6c. During the discharge process, the growth path of discharge products in Li‐CO_2_ battery was changed to the surface‐adsorption growth pathway by enhancing the adsorption energy of the key intermediate Li_2_C_2_O_4_, and finally the thin and uniformly distributed discharge products were obtained. During the charge process, the thin and uniform discharge products are tightly combined with a large number of distributed Ru atoms active sites, making them easier to decompose. In sharp contrast, Li‐CO_2_ batteries based on Co_3_O_4_/CC cathodes generate large bulk products through solvation‐mediated pathway. In this case, the bulk discharge products have poor contact with the active sites, causing them to decompose at higher charging platforms. It should be noted that although the discharge product is solid phase and covers a certain amount of active sites, its influence on the cathode activity is very limited. The reason is that in the process of battery charge and discharge, the active site can not only catalyze the generation/decomposition of discharge products through direct contact but also promote the generation/decomposition of discharge products through the transfer of electrons and holes (hole polarons) with the help of defects in the discharge products, which has also been confirmed by previous reports.^[^ ^58^, ^59^ ^]^

## 3 Conclusion

In conclusion, a facile and eco‐friendly method to prepare SASCs was proposed, and the derived SA Ru‐Co_3_O_4_/CC was successfully applied to Li‐CO_2_ batteries as an efficient dual‐catalyst cathode. Experimental results show that the doped Ru atoms as drive force centers not only improves electrochemical performances of the battery but also tailors the morphology and distribution of discharge products. Critically, DFT simulations demonstrated that SA Ru active site could promote the adsorption of key intermediate (Li_2_C_2_O_4_), thus promoting the discharging process as well as optimizing the growth pathway of discharge products. Additionally, SA Ru active site as an electron‐deficient center also promotes oxidative decomposition of discharge products during charging, and this effectively reduces the charging overpotential. This work not only shares a simple and gentle preparation strategy for the synthesis of SASCs but also provides a new idea for the development of efficient cathode for Li‐CO_2_ batteries.

## Conflict of Interest

The authors declare no conflict of interest.

## Supporting information

Supporting Information

## Data Availability

Data available on request from the authors.
