# Supplementary material for: Single‐Atom Ru Implanted on Co3O4 Nanosheets as Efficient Dual‐Catalyst for Li‐CO2 Batteries
Source: Adv Sci (Weinh). 2021 Oct 20;8(23):2102550. doi: 10.1002/advs.202102550 (PMC8655220; doi:10.1002/advs.202102550)
Supplement: Supplementary file 1 — Supporting Information [file ADVS-8-2102550-s001.pdf]

© 2021 Wiley-VCH GmbH

ADVANCED SCIENCE
Open Access

Supporting Information

for *Adv. Sci.,* DOI: 10.1002/advs.202102550

Single-Atom Ru Implanted on Co_3O_4 Nanosheets as Efficient Dual-Catalyst for Li-CO_2 Batteries

Zheng Lian, Youcai Lu, Chunzhi Wang, Xiaodan Zhu, Shiyu Ma, Zhongjun Li, Qingchao Liu* and Shuangquan Zang*

Mr. Zheng Lian, Dr.Youcai Lu, Ms. Chunzhi Wang, Ms. Xiaodan Zhu, Mr. Shiyu Ma, Prof. Zhongjun Li, Dr. Qingchao Liu and Prof. Shuangquan Zang

College of Chemistry, and Institute of Green Catalysis, Zhengzhou University, Zhengzhou 450001, PR China
Email: qcliu@zzu.edu.cn; lizhongjun@zzu.edu.cn.

1. Experimental section

1.1 Chemicals and Materials.

Carbon cloth (CeTech Co), cobalt nitrate hexahydrate ($Co(NO_3)_2{\cdot}6H_2O$, CAS No. 10026-22-9, AR, 99%; Aladdin), 2-methylimidazole (2-MeIM, $C_4H_6N_2$, CAS No. 693-98-1, 99%; Aladdin), Ruthenium(III) chloride hydrate ($RuCl_3{\cdot}xH_2O$ CAS No. 14898-67-0, AR, 98%; Energy), Deionized water.

1.2 Electrocatalyst preparation

Hydrophilic treatment of CC: Cut the CC into a rectangle about 4×3cm in size. After being bombarded with particles for 3 minutes under Ar plasma (50W), the treated CC became more hydrophilic.

Preparation of Co-MOF/CC: 0.582g cobalt nitrate hexahydrate ($Co(NO_3)_2·6H_2O$) was dissolved in 10mL deionized water to obtain solution A. Solution B was obtained by dissolving 1.314g of dimethylimidazole in 30mL of deionized water. A piece of CC was immersed in solution B and let stand for 5 minutes. Then, A solution was quickly poured into B solution under slight shaking conditions and let stand for two hours. The obtained Co-MOF/CC samples were washed 5 times with deionized water and dried overnight in a 60℃ oven.

Preparation of CoRu-MOF/CC: The obtained Co-MOF/CC was put into $RuCl_3$ aqueous solution with a concentration of 0.3 mg mL^{-1}, and was removed after 40 minutes. Wash with deionized water several times and dry in a 60℃ oven for 12 hours.

Preparation of Co_3O_4/CC: The obtained Co-MOF/CC was put into muffle furnace, rising to 350℃ at a heating rate of 5℃ min^{-1} and kept for 90 minutes. After natural cooling, Co_3O_4/CC was obtained, and no further treatment was required for the obtained samples.

Preparation of SA Ru-Co_3O_4/CC: The obtained CoRu-MOF/CC was put into muffle furnace and heated to 350℃ at 5℃ min^{-1} and kept for 90 minutes. After natural cooling, SA Ru-Co_3O_4/CC was obtained, and no further treatment was required for the obtained samples.

Preparation of RuO_2-Co_3O_4/CC: For the synthesis of the RuO_2-Co_3O_4/CC cathode, the obtained Co_3O_4/CC were immersed into the 3 mg mL^{-1} $RuCl_3$ aqueous solution, then dried in oven at 60 ℃, and finally calcined at 350 ℃ for 2 h in Air atmosphere.

1.3 Materials Characterizations

The morphology of samples was characterized by transmission electron microscopy (a JEM-2100 TEM) and scanning electron microscopy (a Zeiss SIGMA 500 SEM). XPS was

performed on a Thermo escalab 250Xi instrument with Al Ka radiation. The crystal structures of samples were characterized by an X-ray diffraction (XRD, Cu-kα radiation). The Brunauer-Emmet-Teller (BET) surface area was determined by Nitrogen adsorption/desorption isotherms with a TriStar Ⅱ 3020 apparatus at 77 K. Raman spectra were performed on Renishaw in via with a 532 nm laser. Atomic-level high-angle annular dark-field scanning TEM (HAADF-STEM) images were recorded from a probe corrected TEM (JEM-ARM200F) working at 200 kV, coupled with double probe spherical aberration correctors. The X-ray absorption fine structure spectra (Ru K-edge) were collected at BL14W beamline in Shanghai Synchrotron Radiation Facility (SSRF). The storage rings of SSRF was operated at 3.5 GeV with a stable current of 200 mA. Using Si(111) double-crystal monochromator, the data collection were carried out in fluorescence mode using Lytle detector. All spectra were collected in ambient conditions. The XAFS results were fitted *via* the IFEFFIT software. DEMS was performed on i-DEMS 100. The content of the Ru was measured by inductively coupled plasma-optical emission spectrometer (ICP-OES, Thermo Fisher 6500).

1.4 Assembly and testing of Li-CO_2 batteries

The electrochemical performances of the Li-CO_2 batteries were tested in a 2025-type coin cell. All of the batteries were assembled in a glove box in an Ar atmosphere with a lithium foil anode, a glass fibre separator, a cathode and an electrolyte containing 1 M LiTFSI in TEGDME. Note that, the original cathodes can be used directly by simple clipping without any processing. The active material loading was ~0.5 mg cm^{-2}. The electrochemical performances of the Co_3O_4/CC and SA Ru-Co_3O_4/CC cathode catalysts were tested in a specific capacity-controlled mode under various current densities. For comparison, clean CC was also considered as a cathode. Then transfer the prepared battery to a sealed glass bottle filled with CO_2, and perform galvanostatic charge-discharge cycles on a LAND CT2001A multichannel battery system.

1.5 Theoretical Calculations

The Vienna Ab Initio Package (VASP).[1-3] was employed to perform all the spin-polarized density functional theory (DFT) calculations within the generalized gradient approximation (GGA) using the Perdew-Burke-Ernzerhof (PBE) formulation.[4] The Hubbard U (DFT+U) corrections of transition metals (3.42/Co, 2.42/Ru) were considered in the calculations.[5] The projected augmented wave (PAW) potentials[6] was chosen to describe the ionic cores and take valence electrons into account using a plane wave basis set with a kinetic energy cutoff of 500 eV. Partial occupancies of the Kohn-Sham orbitals were allowed using the Gaussian smearing method and a width of 0.10 eV. The electronic energy was considered self-consistent when the energy change was smaller than 10^{-4} eV. The maximum Hellmann-Feynman force for each ionic optimization step is 0.05eV $Å^{-1}$, as well as the optimization of equilibrium lattice constants. The configurations of catalysts are established, named Co_3O_4 and SA Ru-Co_3O_4. A p(4x4) unit cell was chose for both catalysts, and six layers were built in their slab configuration. This slab was separated by a 20 Å vacuum layer in the z direction between the slab and its periodic images. A 3×3×1 Monkhorst-Pack k-point grid for Brillouin zone sampling was used in structural optimization and energy calculation. In order to fully consider the activity of each catalyst, the possible catalytic sites in catalysts are selected to discuss their adsorption behavior. The free energy (ΔG) of Li-CO_2 battery on those catalysts was defined as[1] $\Delta G = E_{DFT} + E_{ZPE} - T \times S + \int CpdT$ (1), where E_{DFT}, E_{ZPE}, T, S and Cp are the calculation total energy of intermediate on catalysts surface, the zero-point energy, temperature, entropy and heat capacity. The over-potential ($\eta^{discharge}$) of Li-CO_2 battery was defined as: $\eta^{discharge} = max\{\Delta G_1, \Delta G_2, \Delta G_3, \Delta G_4, \Delta G_5, \Delta G_6, \Delta G_7, \Delta G_8\}$, Where the ΔG1, ΔG2, ΔG3, ΔG4, ΔG5, ΔG6, ΔG7, ΔG8 are the free energy for four elementary reactions of Li-CO_2 battery (Figure 5a).

2. Supplementary Figures

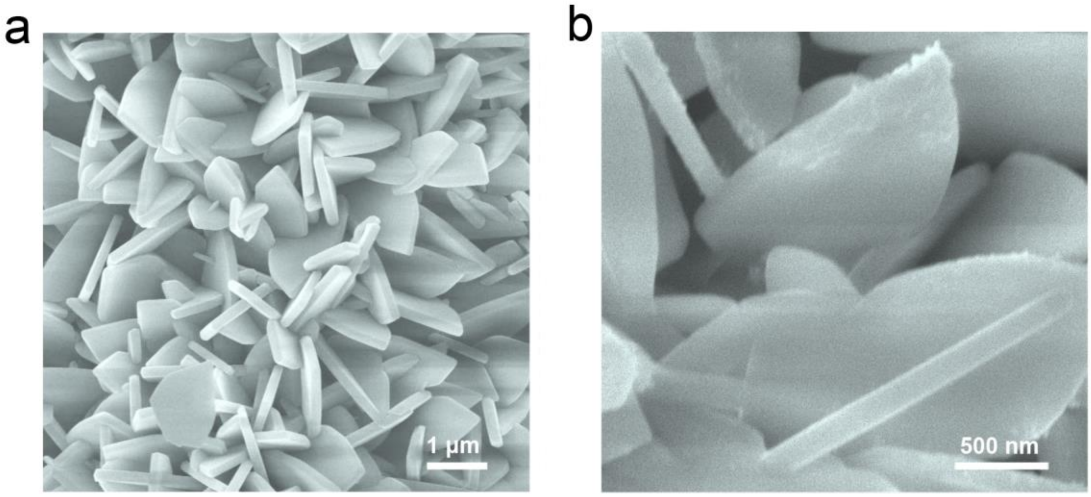

Figure S1. (a) SEM image of Co-MOF/CC. (b) SEM image of Co-MOF/CC etched by HCl, the concentration of HCl solution was about 1 mM.

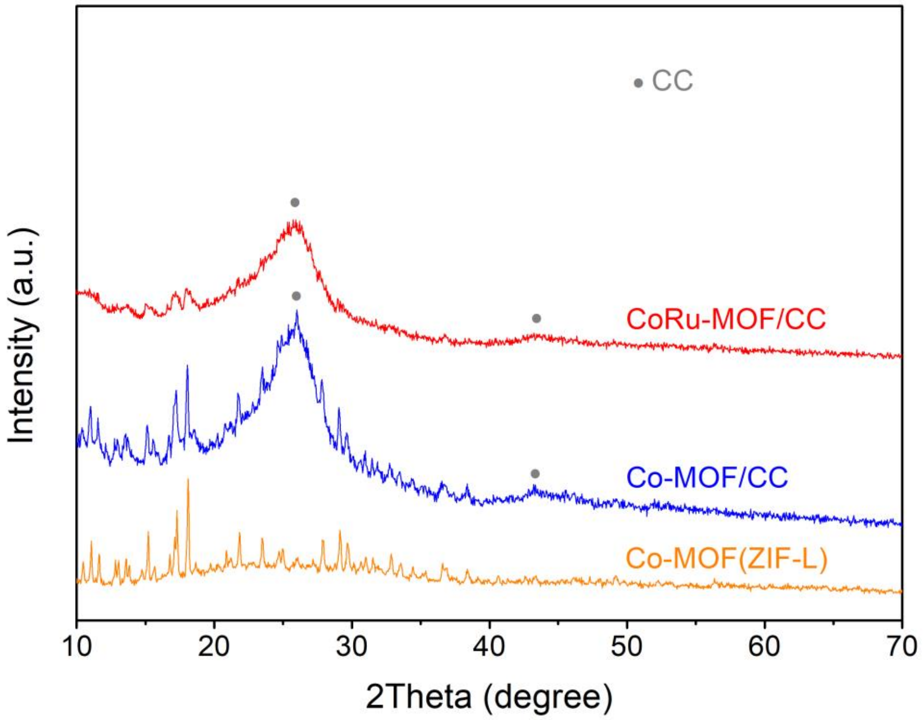

Figure S2. XRD patterns of pure Co-MOF (ZIF-L), Co-MOF/CC and CoRu-MOF/CC.

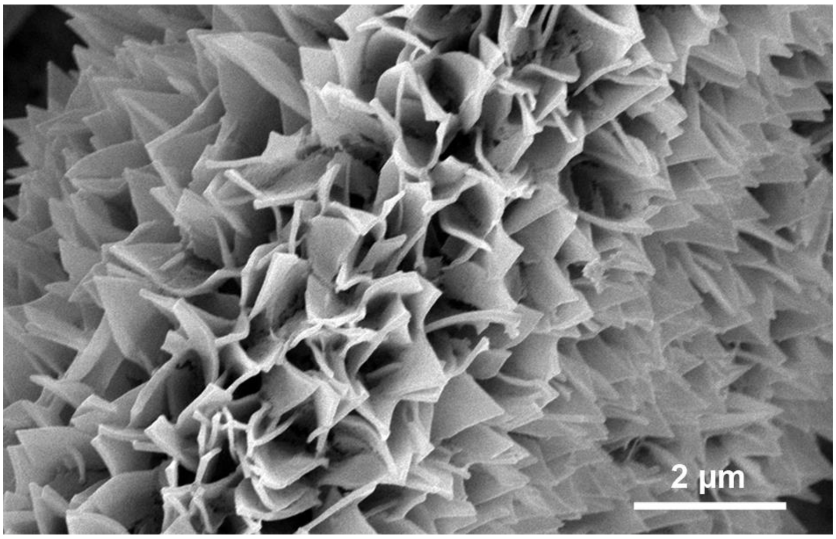

Figure S3. SEM image of Co_3O_4/CC,.

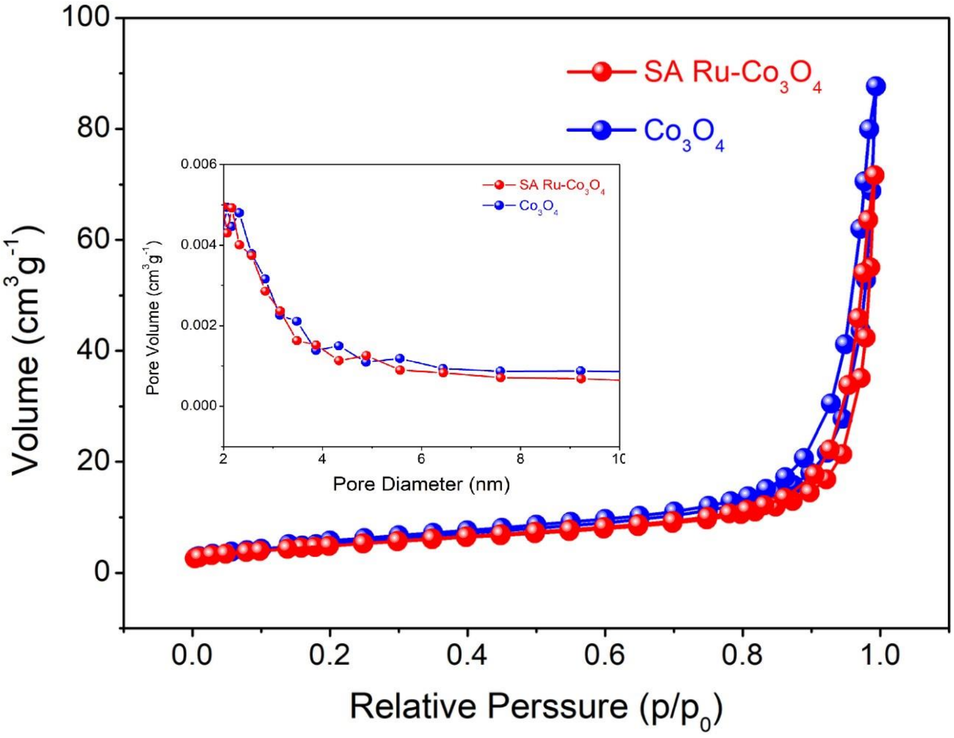

Figure S4. Nitrogen adsorption/desorption isotherms and related pore size distribution (the insert) of Co_3O and SA Ru-Co_3O_4.

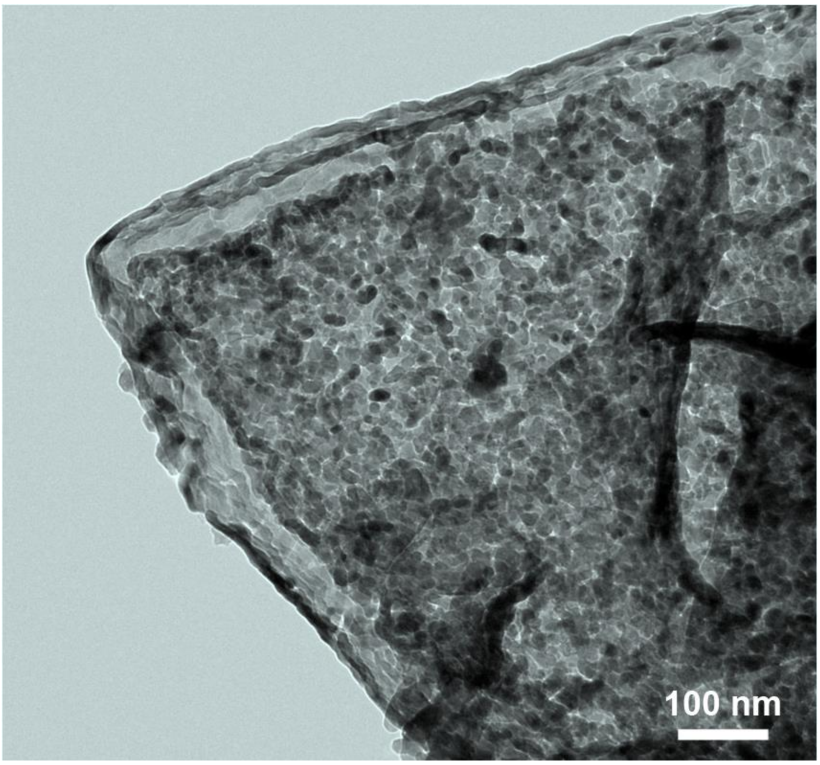

Figure S5. TEM image of Co_3O_4 nanosheet.

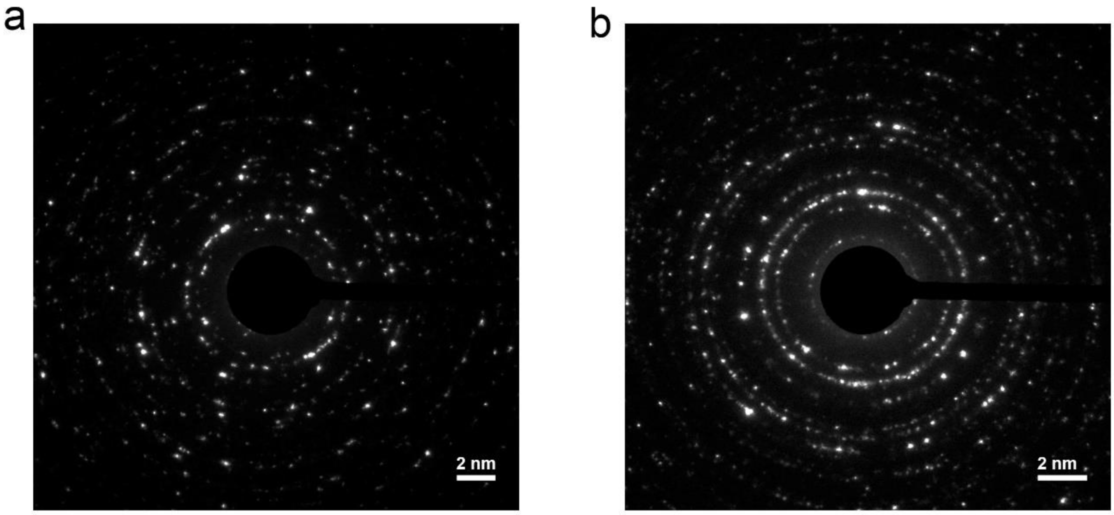

Figure S6. Selected area electron diffraction (SAED) pattern of (a) Co_3O_4, (b) SA Ru-Co_3O_4.

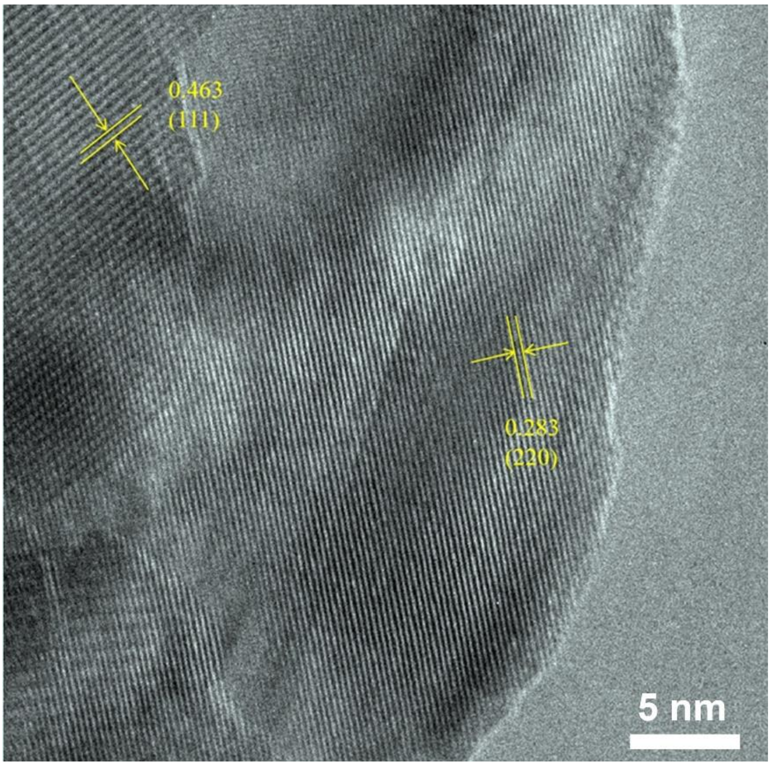

Figure S7. HR-TEM image of Co_3O_4.

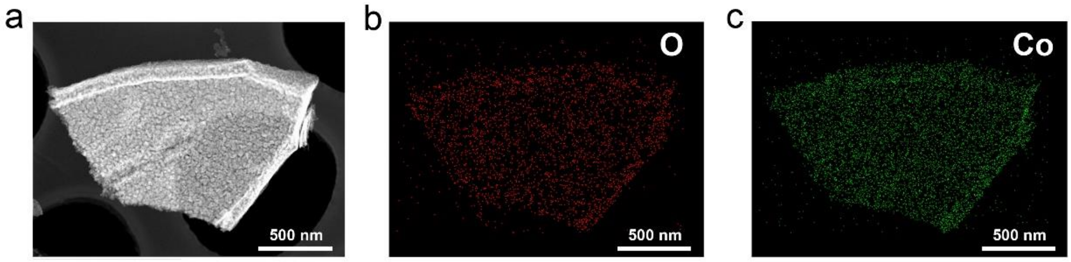

Figure S8. (a) TEM image of Co_3O_4 nanosheet, and the corresponding (b) O element and (c) Co element.

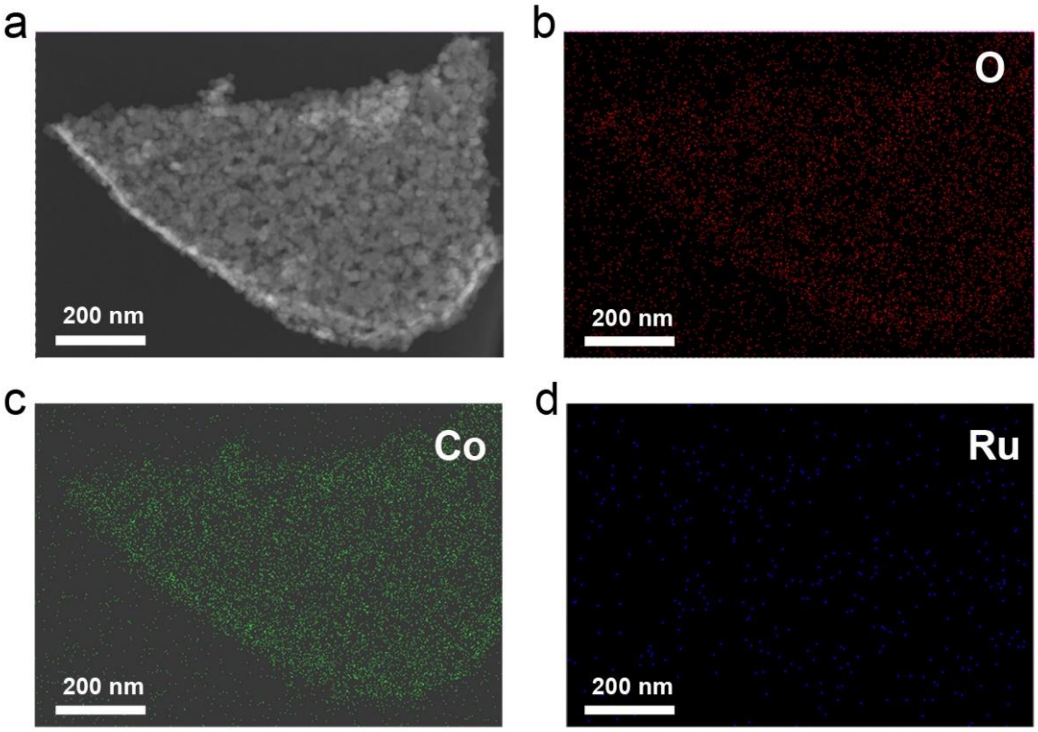

Figure S9. (a) TEM image of SA Ru-Co_3O_4 nanosheet, and the corresponding (b) O element, (c) Co element and (d) Ru element.

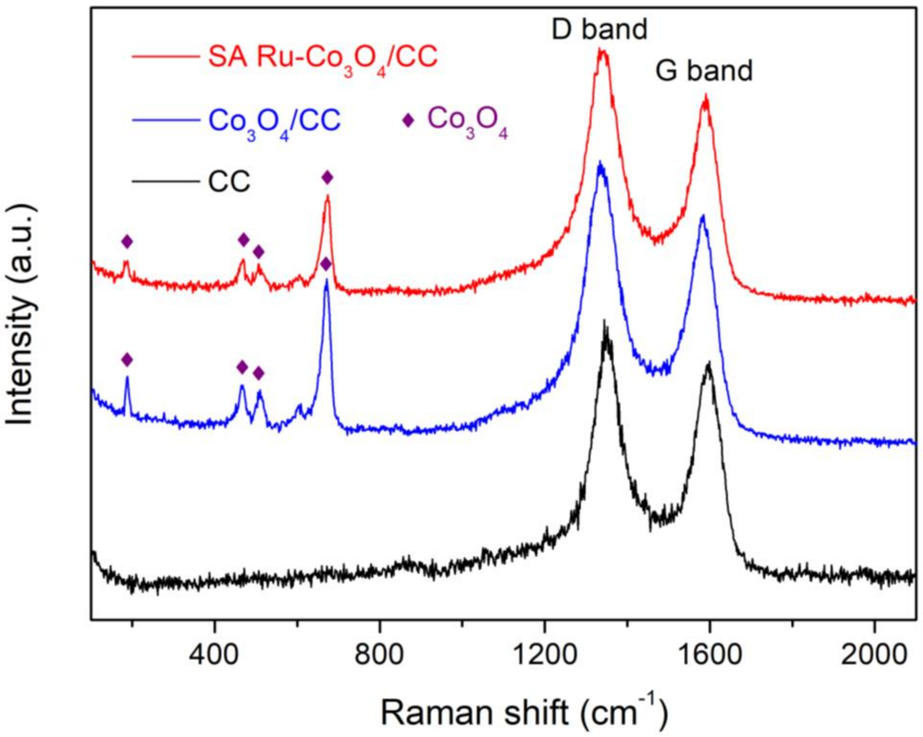

Figure S10. Raman spectra of CC, Co_3O_4/CC and SA Ru-Co_3O_4/CC.

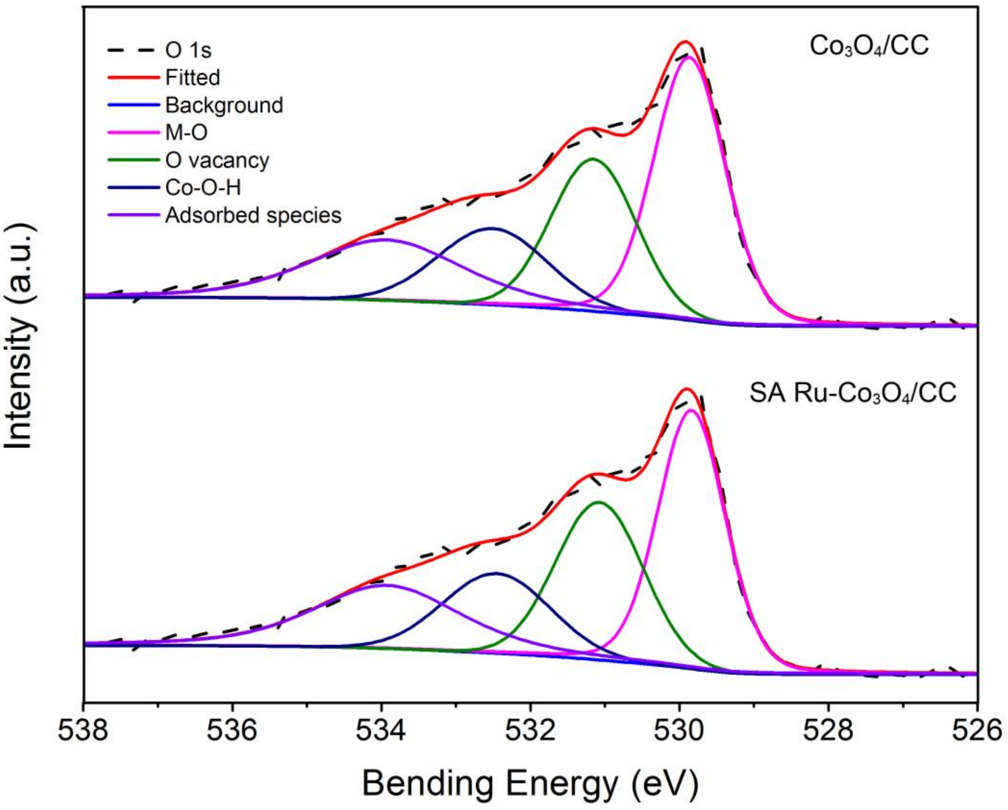

Figure S11. XPS spectra of O 1s of Co_3O_4/CC and SA Ru-Co_3O_4/CC.

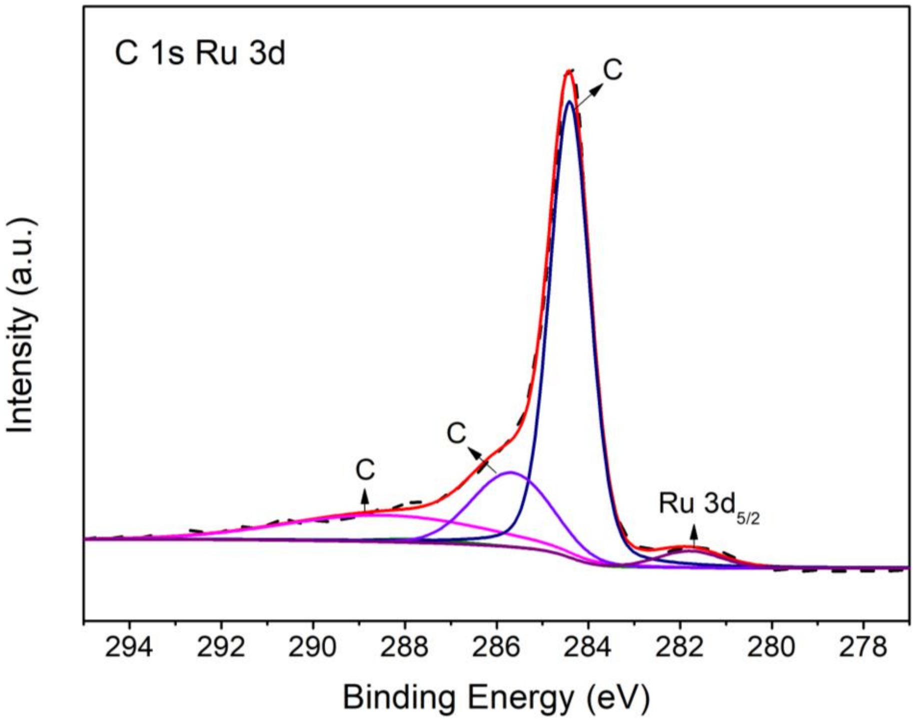

Figure S12. High-resolution XPS spectra of C 1s and Ru 3d of SA Ru-Co_3O_4/CC.

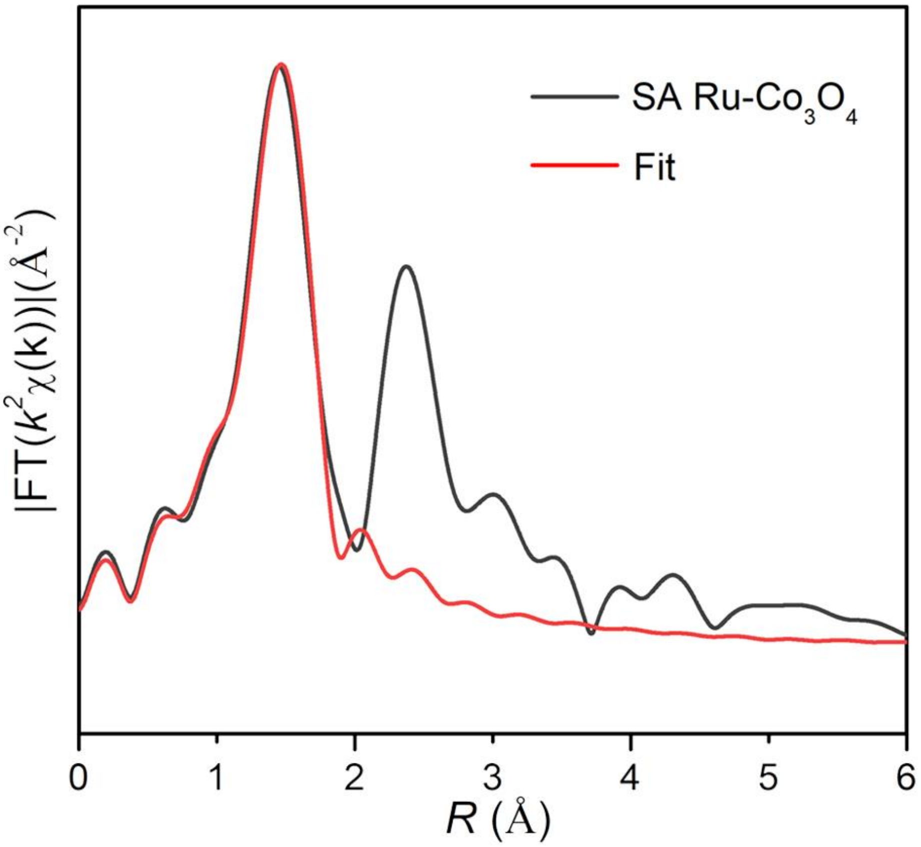

Figure S13. Fourier-transformed magnitudes of Ru K-edge EXAFS spectra in R space for SA $Ru-Co_3O_4$ sample.

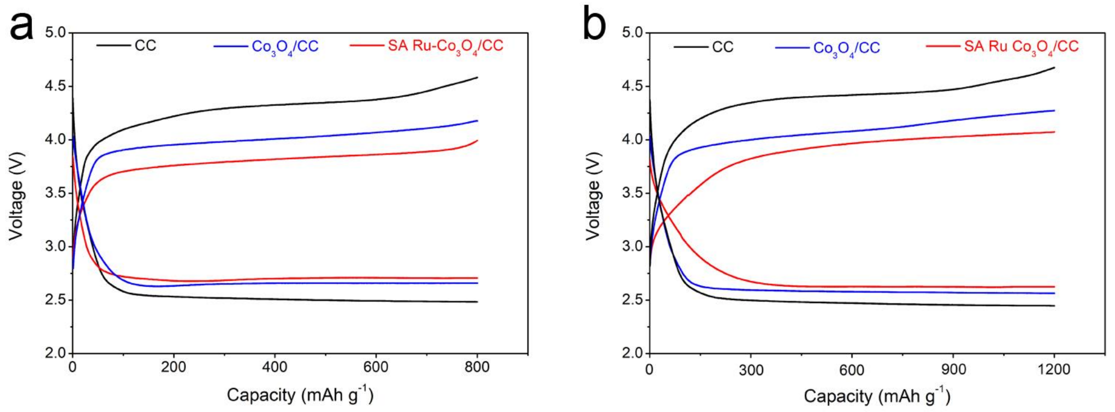

Figure S14. (a) Initial discharge/charge profiles of CC, Co_3O_4/CC and SA Ru-Co_3O_4/CC at 200 mA g^{-1} under the limited discharge/charge capacities of 800 mAh g^{-1}. (b) Initial discharge/charge profile of CC, Co_3O_4/CC and SA Ru-Co_3O_4/CC at 300 mA g^{-1} under the limited capacities of 1200 mAh g^{-1}.

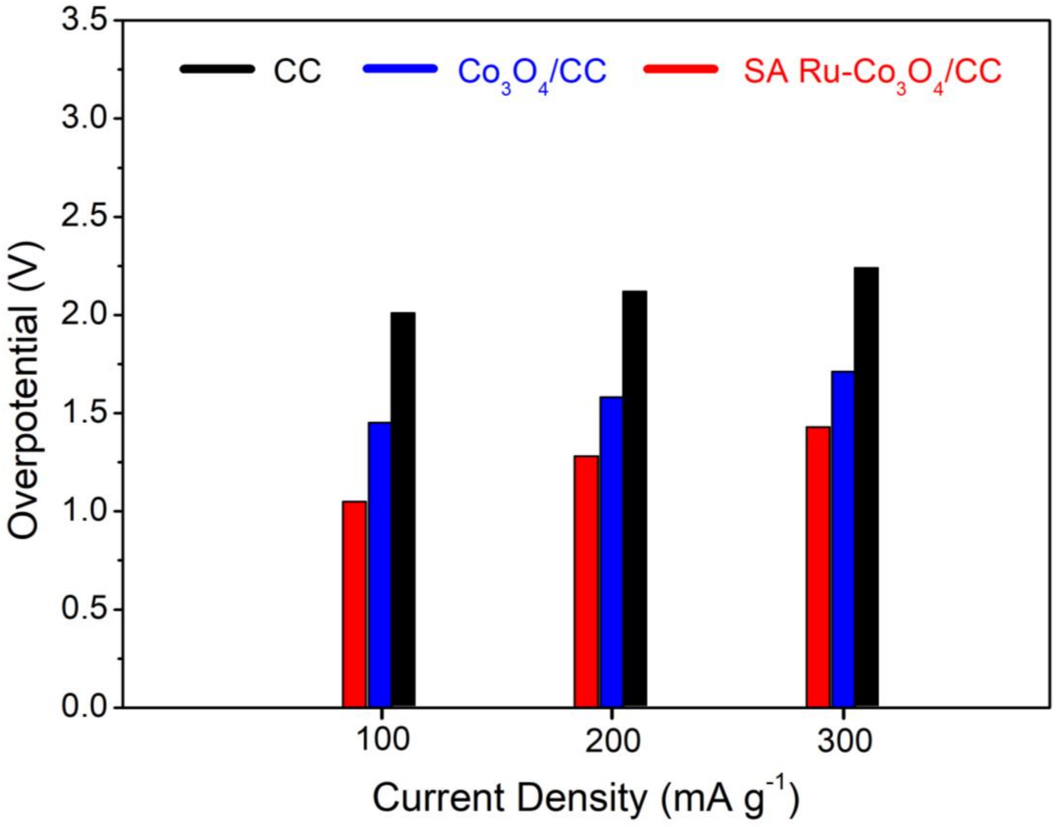

Figure S15. The over-potential profiles of CC, Co_3O_4/CC and SA Ru-Co_3O_4/CC at different current density.

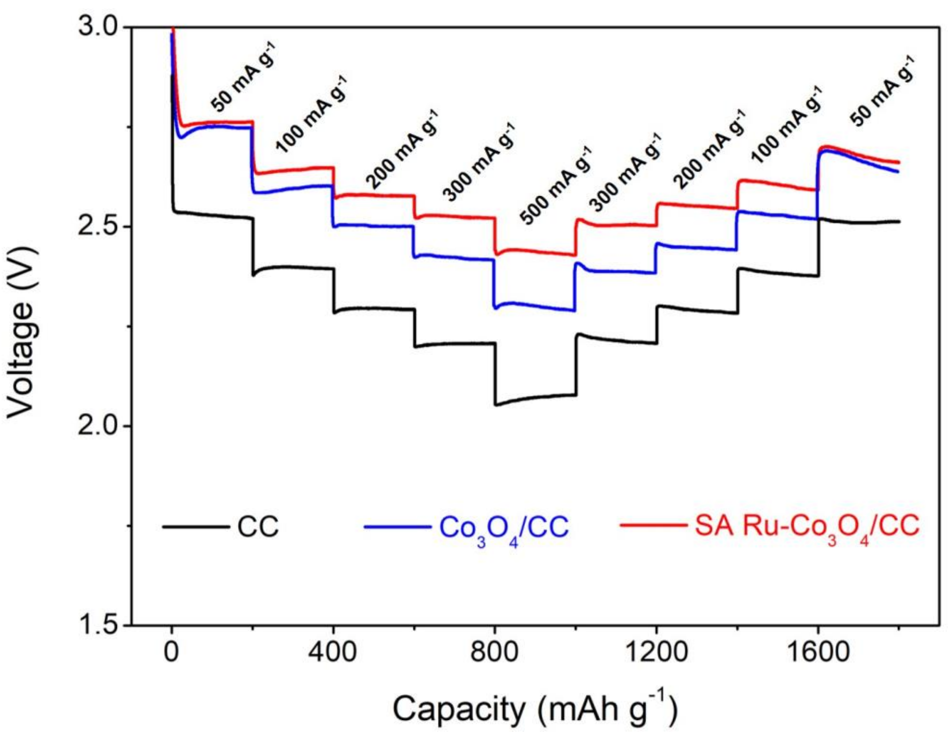

Figure S16. The rate performance of CC, Co_3O_4/CC and SA Ru-Co_3O_4/CC, the current density ranges from 50 to 500 mA g^{-1}.

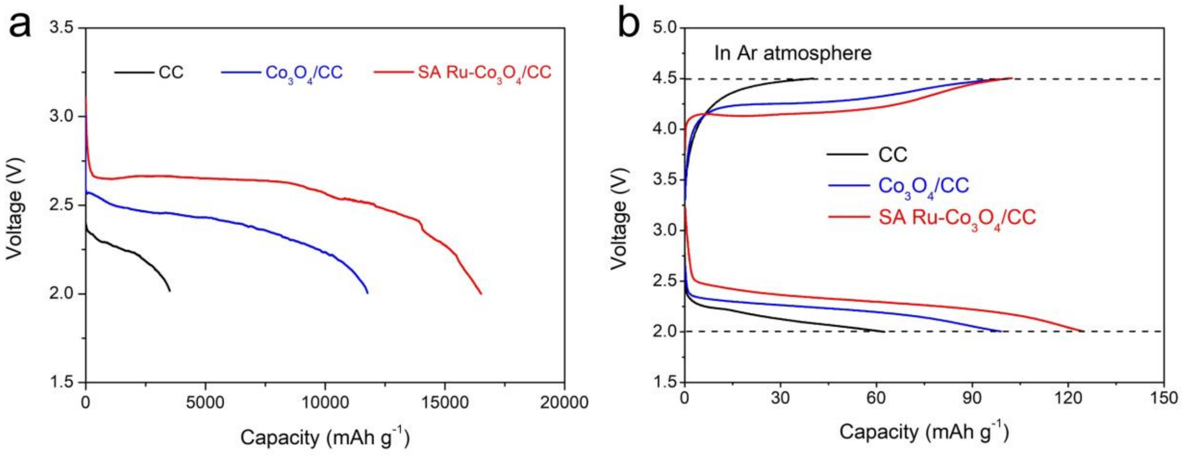

Figure S17. (a) Full discharge profiles of CC, Co_3O_4/CC and SA Ru-Co_3O_4/CC tested at 300 mA g^{-1}. (b) Discharg-charge curves of Li-CO_2 batteries with different cathdoes under Ar atmosphere.

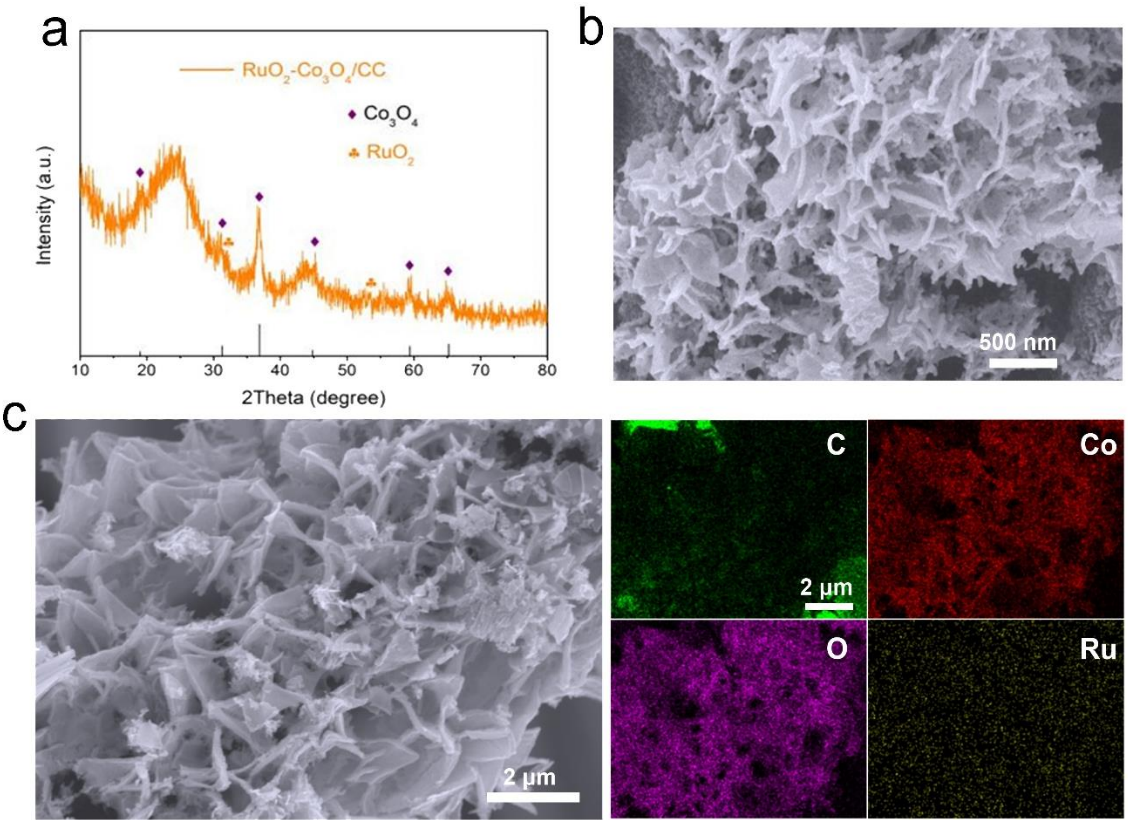

Figure S18. (a, b) XRD pattern and SEM image of RuO_2-Co_3O_4/CC cathode (c) SEM and the corresponding elemental mapping images of RuO_2-Co_3O_4/CC.

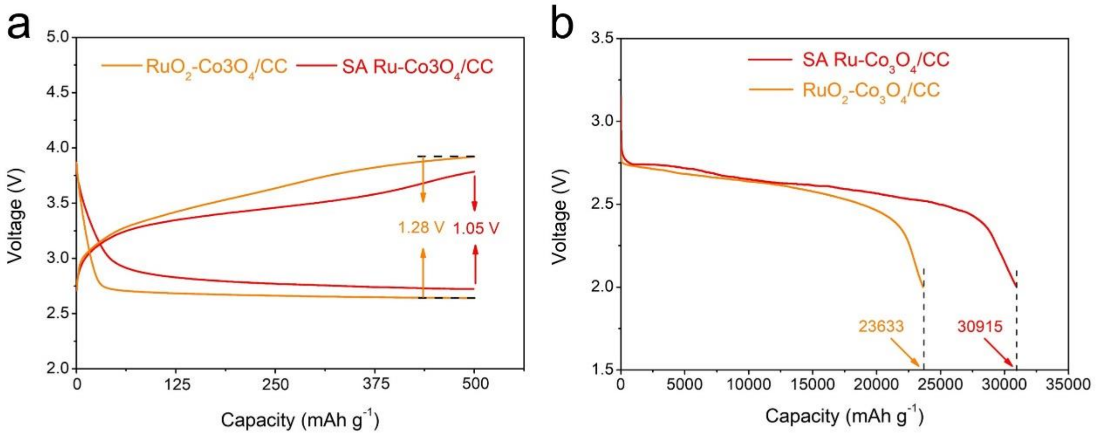

Figure S19. (a) First cycle curves Li-CO_2 batteries with different cathodes under the limited capacity of 500 mAh g^{-1} with current density of 100 mA g^{-1}. (b) Depth discharge capacity of Li-CO_2 batteries based on different cathodes with the current density of 100 mA g^{-1}.

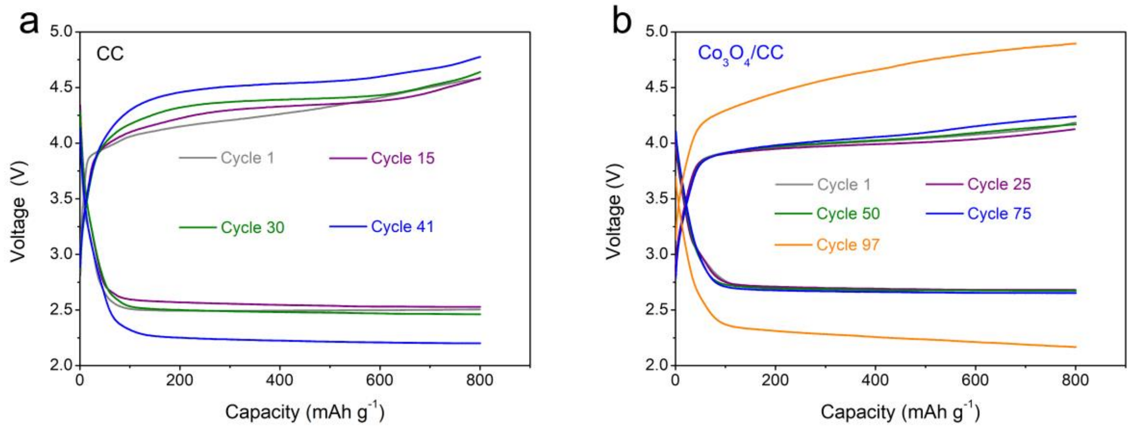

Figure S20. Discharge/charge profiles tested at 200 mA g^{-1} of (a) CC and (b) Co_3O_4/CC cathodes.

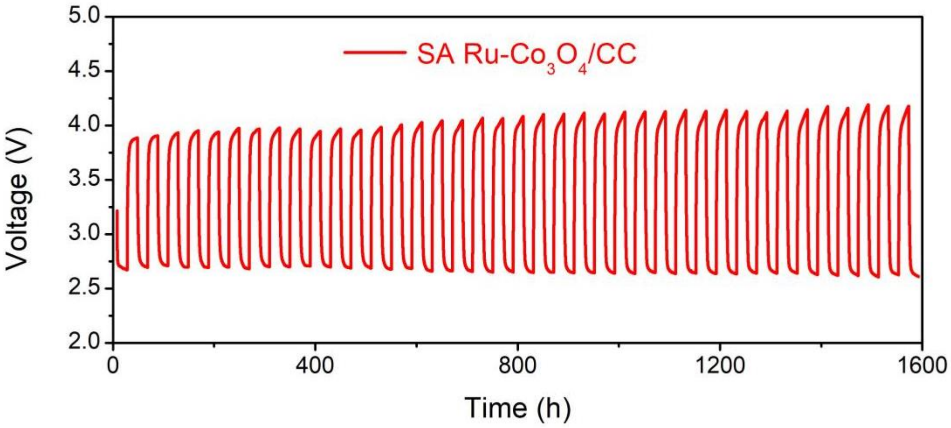

Figure 21. Cycle performance of Li-CO_2 battery based on SA Ru-Co_3O_4/CC cathode under the limited capacity of 2000 mAh g^{-1} with current density of 100 mA g^{-1}.

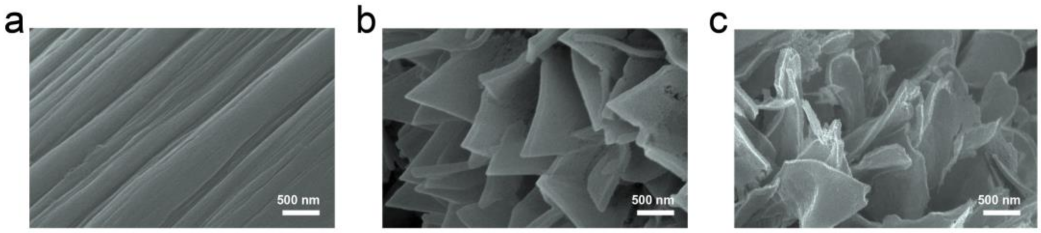

Figure S22. SEM images of (a) pristine CC, (b) Co_3O_4/CC and (c) SA Ru-Co_3O_4/CC cathodes.

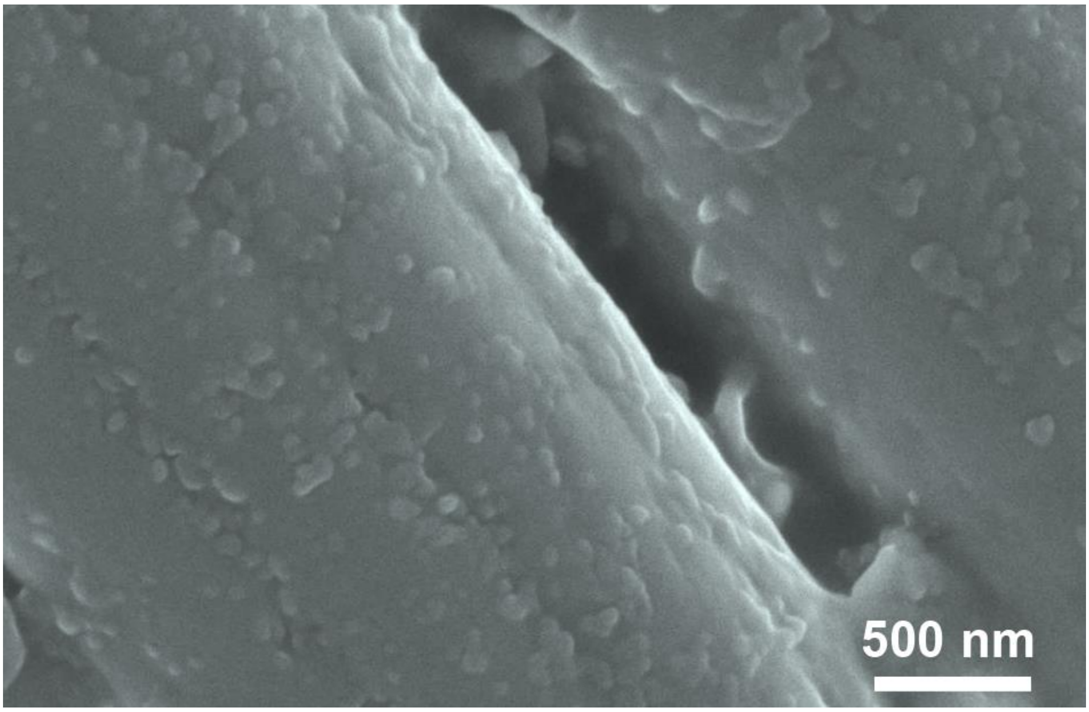

Figure S23. SEM image of CC cathode after 5 cycles.

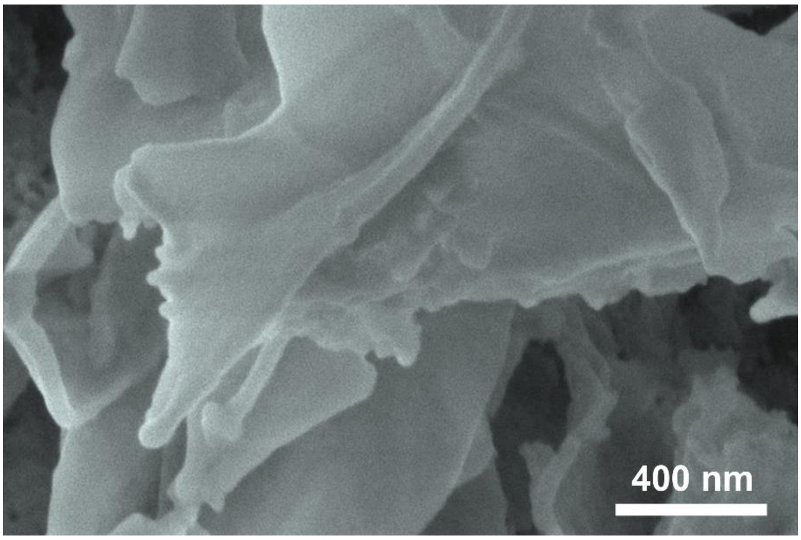

Figure S24. The SEM image of Co_3O_4/CC cathode after 5 cycles.

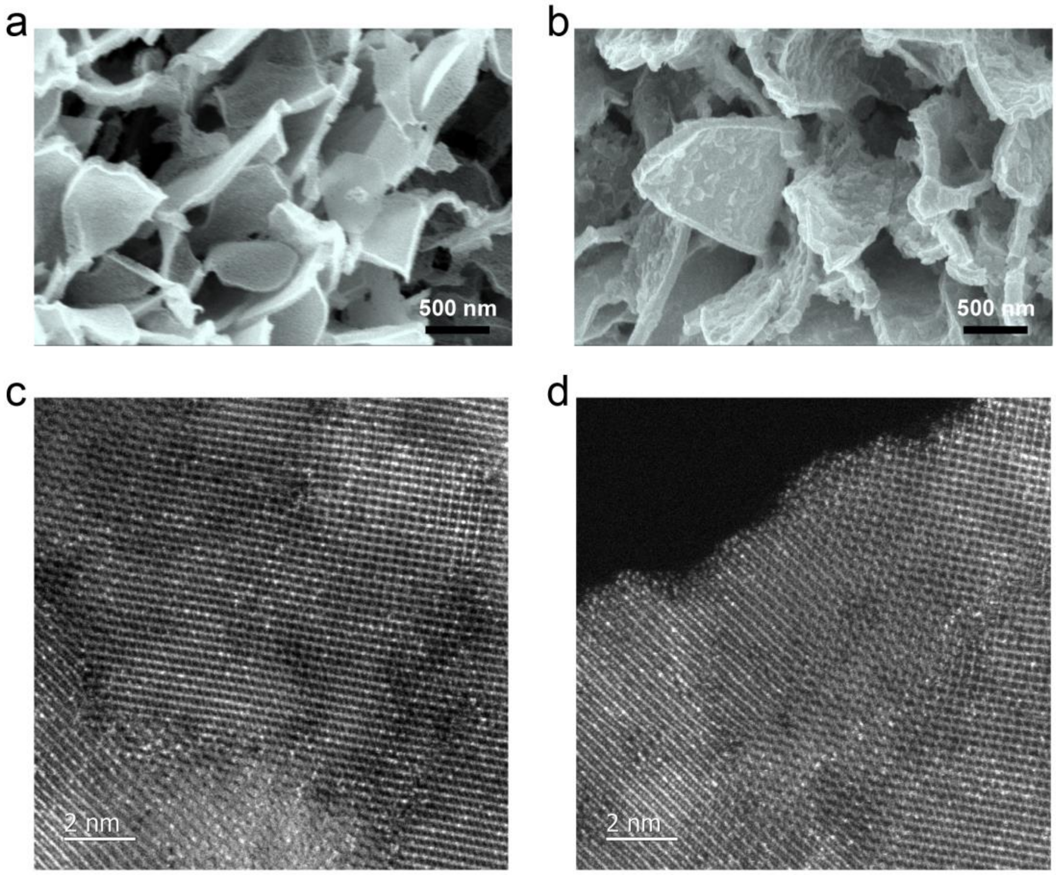

Figure S25. SEM image of SA Ru-Co_3O_4/CC cathode (a) after 5 cycles and (b) after 30 cycles.

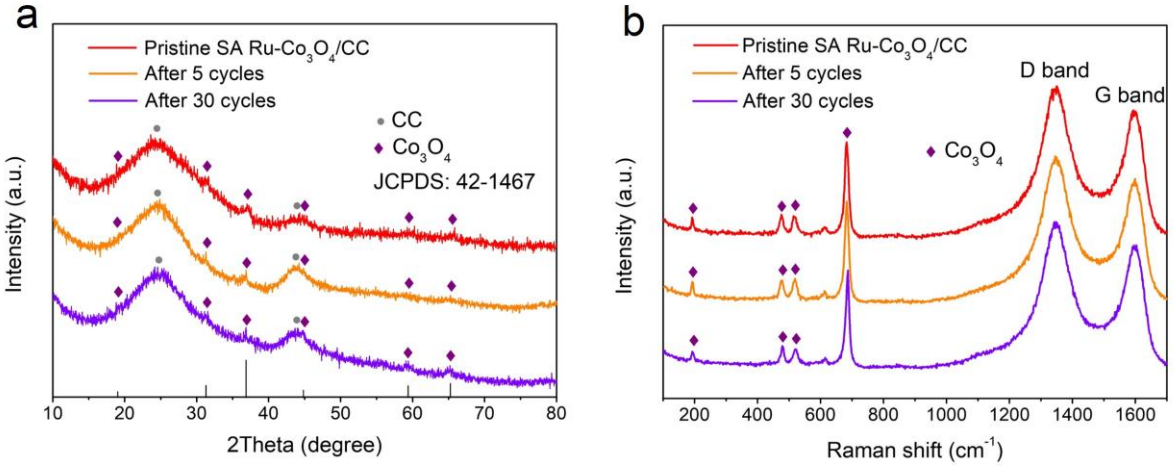

Figure 26. (a) XRD patterns (b) Raman spectra of SA Ru-Co_3O_4/CC cathode with different cycles.

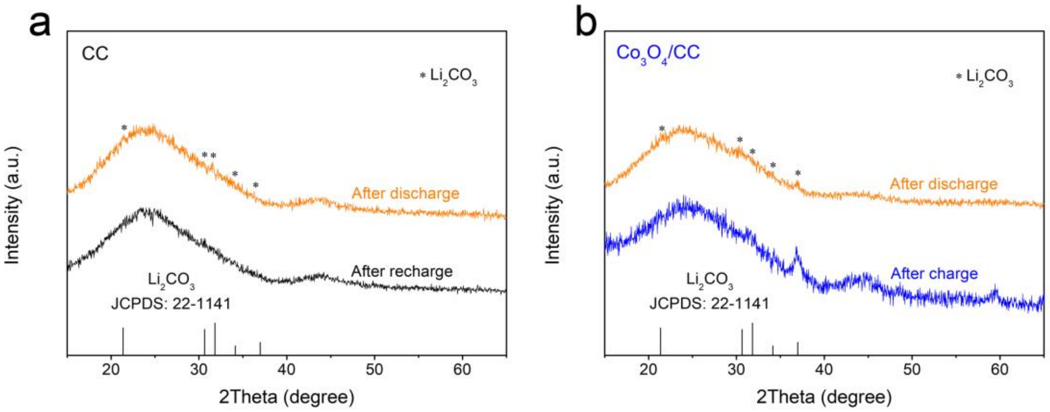

Figure S27. XRD patterns of (a) CC and (b) Co_3O_4/CC cathodes under discharged and recharged states.

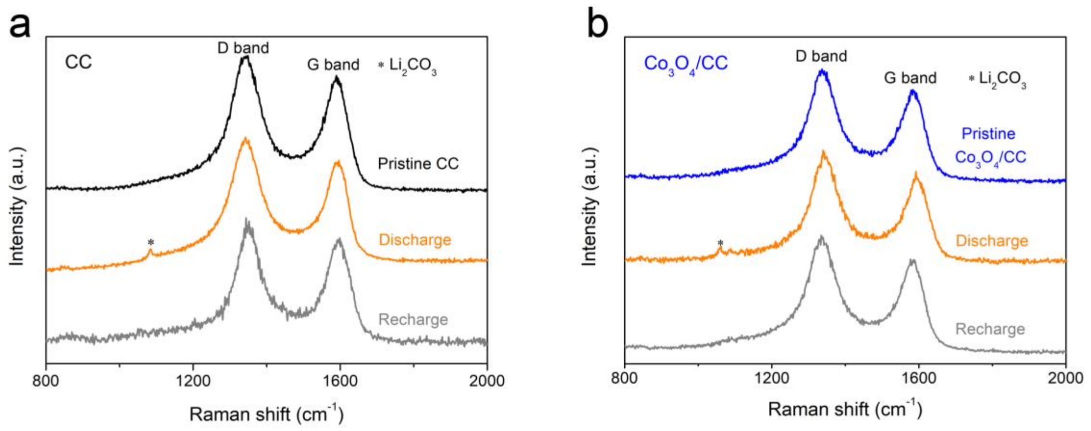

Figure S28. Raman spectra of (a) CC and (b) Co_3O_4/CC cathodes under pristine, discharged and recharged states.

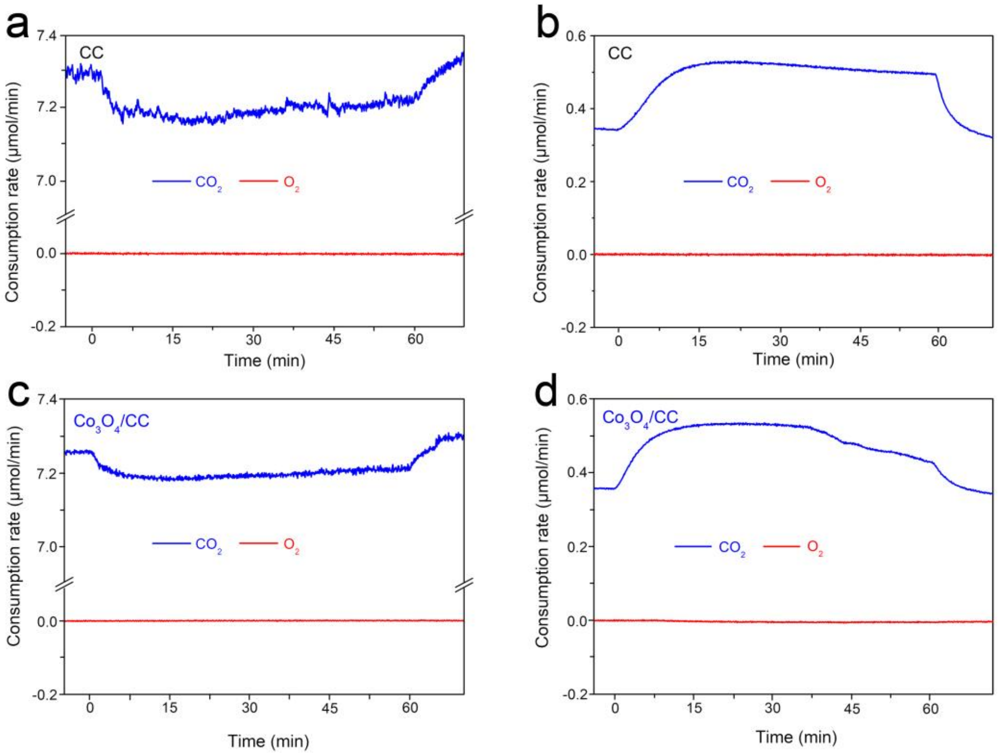

Figure S29. (a) DEMS test of Li-CO_2 battery based a CC cathode during discharging. (b) DEMS image of Li-CO_2 battery based a CC cathode during charging. (c) DEMS image of Li-CO_2 battery based a Co_3O_4/CC cathode during discharging. (d) DEMS image of Li-CO_2 battery based a Co_3O_4/CC cathode during charging.

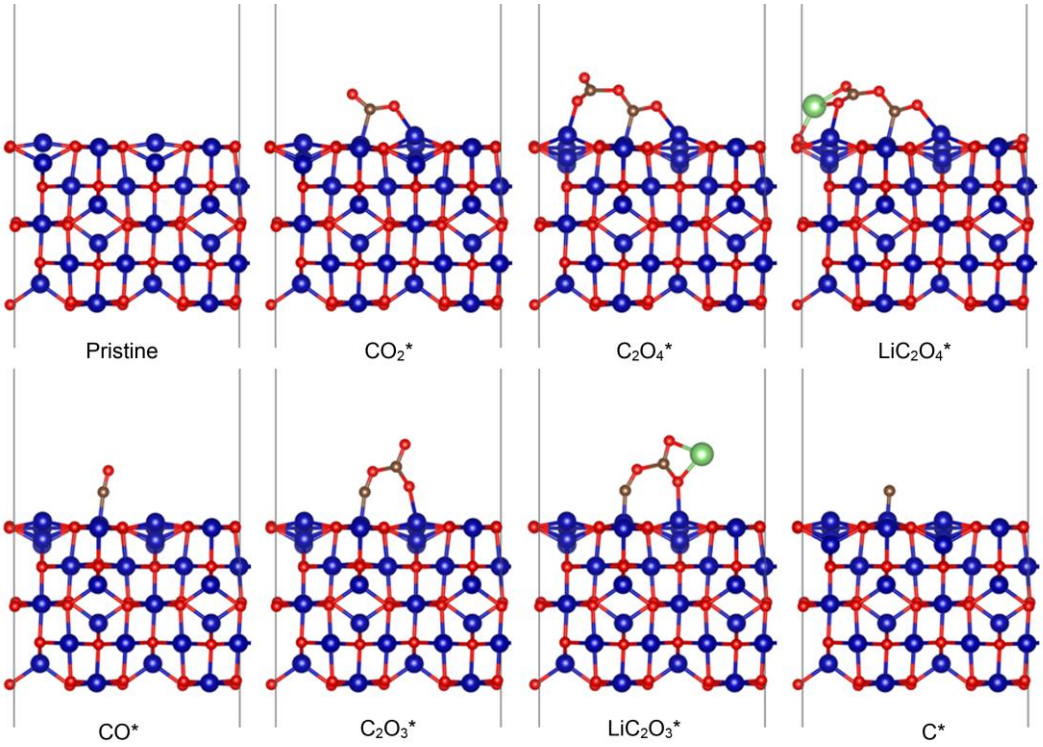

Figure S30. Simulation of reactants and reaction intermediates on the "Co_3O_4" active sites during discharge.

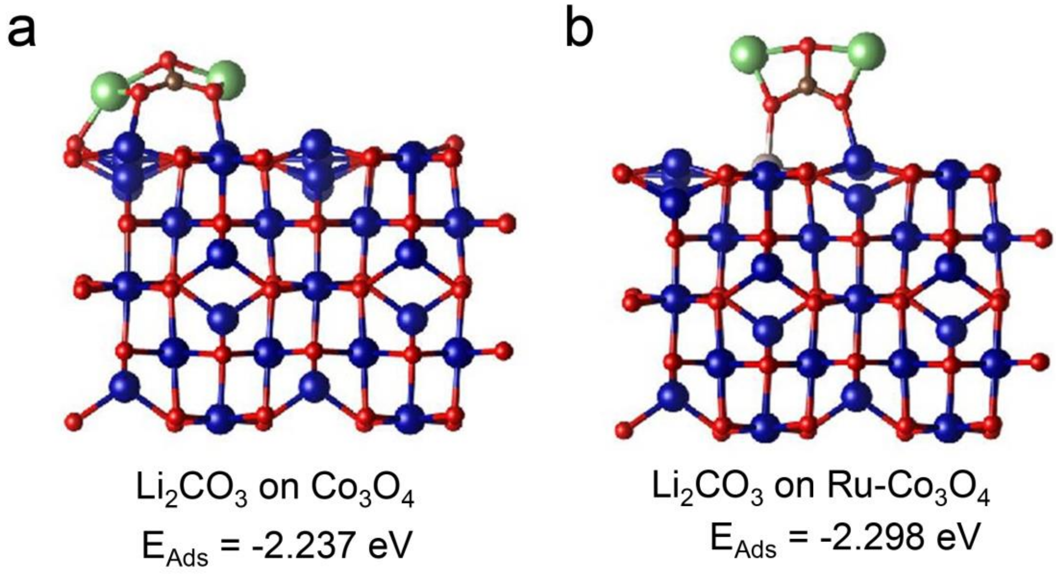

Figure S31. Adsorption energy of reaction intermediate Li_2CO_3 on (a) "Co_3O_4" and (b) "SA Ru-Co_3O_4" active sites.

3. Supplementary Tables

Table S1. Structural parameters extracted from the Ru K-edge EXAFS fitting.

Sample	Path	CN	R(Å)	$\sigma^2(10^{-3}Å^2)$	ΔE_0 (eV)	R-factor
SA Ru-Co_3O_4	Ru-O	5.0 ± 0.3	2.00 ± 0.02	4.6 ± 0.8	-1.9 ± 0.9	0.005

CN, coordination number;

R, bonding distance;

σ^2, Debye-Waller factor;

ΔE_0, inner potential shift.

Table S2. Comparison of electrochemical performances of Li-CO_2 batteries with different cathodes.

Cathode	Current (mA g^{-1})	Capacity (mAh g^{-1})	Cycle Capacity (mAh g^{-1})	Cycle Number	Over potential (V)	Ref.
adjacent Co/GO	100	17358	1000	100	1.42	7
Fe-ISA/N,S-HG	100	23174	1000	200	1.17	8
RuO_2-TiO_2 NAs/CT	250	16727	1000	238	1.05	9
Ru/Ni	100	9502	1000	100	1.20	10
Ru/NS-G	100	12448	1000	100	1.13	11
IrO_2-N/CNT	100	4634	400	316	1.34	12
RuP_2-NPCFs	200	11951	500	200	1.28	13
Mn_2O_3-Mn_3O_4	100	19024	1000	69	1.24	14
MnO@NC-G	50	25021	1000	200	0.88	15
$ZnCo_2O_4$@CNTs	100	4275	500	230	1.7	16
**SA Ru-Co_3O_4/CC**	**200**	**30915**	**800**	**251**	**1.05**	**This work**

Table S3. Adsorption energy of reactant CO_2, key reaction intermediate $Li_2C_2O_4$ and discharge product Li_2CO_3 on the active sites of "Co_3O_4" and "SA Ru-Co_3O_4".

Adsorption energy	CO_2	Li_2CO_3	$Li_2C_2O_4$
Co_3O_4	0.17 ev	-2.237 eV	-2.836 eV
SA Ru-Co_3O_4	1.9 eV	-2.298 eV	-4.927 eV

Table S4. The changes of bond length and angle of CO_2 on the active sites "Co_3O_4" and "SA Ru-Co_3O_4".

	CO_2	CO_2 on Co_3O_4	CO_2 on SA Ru-Co_3O_4
Bond length	1.16 Å	1.219-1.289 Å	1.243-1.240 Å
Bond angle	180°	132.01°	141.77°

Supplementary References

[1] C. G. Hu, L. L. Gong, Y. Xiao, Y. F. Yuan, N. M. Bedford, Z. H. Xia, L. Ma, T. P. Wu, Y. Lin, J. W. Connell, R. Shahbazian-Yassar, J. Lu, K. Amine, L. M. Dai, *Adv. Mater.* **2020**, 32, 1907436.

[2] G. Kresse, J. Furthmüller, Comput. *Mater. Sci.* **1996**, 6, 15.

[3] G. Kresse, J. Furthmüller, *Phys. Rev. B* **1996**, 54, 11169.

[4] J. P. Perdew, K. Burke, *Phys. Rev. Lett.* **1996**, 77, 3865.

[5] L. Gong, X. Wang, T. Zheng, J. Liu, J. Wang, Y. C. Yang, J. Zhang, X. Han, L. Zhang, Z. Xia, *J. Mater. Chem. A* **2021**, 9, 3555.

[6] G. Kresse, D. Joubert, *Phys. Rev. B* **1999**, 59, 1758.

[7] B. W. Zhang, Y. Jiao, D. L. Chao, C. Ye, Y. X. Wang, K. Davey, H. K. Liu, S. X. Dou, S.

Z. Qiao, *Adv. Funct. Mater.* **2019**, 29, 1904206.

[8] C. G. Hu, L. L. Gong, Y. Xiao, Y. F. Yuan, N. M. Bedford, Z. H. Xia, L. Ma, T. P. Wu, Y. Lin, J. W. Connell, R. Shahbazian‐Yassar, J. Lu, K. Amine, L. M. Dai, *Adv. Mater.* **2020**, 32, 1907436.

[9] C. Z. Wang, Y. Shang, Y. C. Lu, L. B. Qu, H. C. Yao, Z. J. Li, Q. C. Liu, *J. Power Sources.* **2020**, 475, 228703.

[10] H. M. Zhao, D. D. Li,H. D. Li, A. G. Tamirat, X. Y. Song, Z. X. Zhang, Y. G. Wang, Z. Y. Guo, L. Wang, S. H. Feng, *Electrochim. Acta* **2019**, 299, 592.

[11] Y. Qiao, J. W. Wu, J. Zhao, Q. L. Li, P. J. Zhang, C. S. Hai, X. L. Liu, S. T. Yang, Y. Liu, *Energy Storage Mater.* **2020**, 27, 133.

[12] G. Wu, X. Li, Z. Zhang, P. Dong, M. L. Xu, H. L. Peng, X. Y. Zeng, Y. J. Zhang, S. J. Liao, *J. Mater. Chem. A* **2020**, 8, 3763.

[13] Z. Y. Guo, J. L. Li, H. C. Qi, X. M. Su, H. D. Li, A. G. Tamirat, J. Liu, Y. G. Wang, L. Wang, *Small* **2019**, 15, 1803246.

[14] L. M. Liu, L. B. Zhang, K. Wang, H. Wu, H. Mao, L. Li, Z. J. Sun, S. Y. Lu, D. Y. Zhang, W. Yu, S. J. Ding, *ACS Appl. Mater. Interfaces* **2020**, 12, 33846.

[15] S. W. Li, Y. Liu, J. W. Zhou, S. S. Hong, Y. Dong, J. M. Wang, X. Gao, P. F. Qi, Y. Z. Han, B. Wang, *Energy Environ. Sci.* **2019**, 12, 1046.

[16] S. Thoka, C. J. Chen, A. Jena, F. M. Wang, X. C. Wang, H. Chang, S. F. Hu, R. S. Liu, *ACS Appl. Mater. Interfaces* **2020**, 12, 17353.
